# Family collective agency in career decision-making among medical and dental students: a qualitative intersectional study in Peshawar, Pakistan

**DOI:** 10.3389/fmed.2026.1755128

**Published:** 2026-02-10

**Authors:** Sadia Qazi, Abdal Ahmad, Syed Ibadullah Shah, Ayat Ullah Khan, Muhammad Umair Amjad, Anas Muiz Jehan, Danyal Ahmad, Gul Saman, Jalwa Rahman, Eshal Atif, Muhammad Atif Mazhar

**Affiliations:** 1 Department of Anatomy, College of Medicine, Alfaisal University, Riyadh, Saudi Arabia; 2 Peshawar Medical College, Riphah International University, Peshawar Campus, Peshawar, Pakistan; 3 Department of Medicine, Bacha Khan Medical Complex, Swabi, Pakistan; 4 College of Medicine, Alfaisal University, Riyadh, Saudi Arabia

**Keywords:** career decision-making, clinical rotations, collectivist culture, Communities of Practice, dental students, family collective agency, gender barriers, intersectionality

## Abstract

**Background:**

Career decision-making in medical education predominantly reflects Western individualistic frameworks emphasizing personal autonomy. The intersection of clinical rotation experiences with family collective agency and culturally embedded gender norms in collectivist contexts remains insufficiently examined.

**Methods:**

We conducted 32 semi-structured interviews with medical (*n* = 18; 9 female, 9 male) and dental students (*n* = 14; 8 female, 6 male) aged 20–25 years across four institutions in Peshawar, Pakistan (August–November 2024). We used reflexive thematic analysis, applying Social Cognitive Career Theory (SCCT), Communities of Practice (CoP), and intersectionality as sensitizing concepts to examine how rotation experiences are interpreted through family decision-making and Pashtun sociocultural norms.

**Results:**

Five interrelated themes emerged. Clinical stress was frequently interpreted through family honor (*izzat*), extending beyond individual performance concerns. Career decision-making operated through collective family deliberation, with economic obligation commonly shaping men’s specialty trajectories and marriage-related expectations consistently constraining women’s career planning through anticipatory specialty screening. Participation patterns ranged from progressive apprenticeship to persistent gender-based marginalization, including exclusion of female students from male patient examinations and restricted access to night shifts in Peshawar institutions. Female participants described a “doctor daughter” paradox in which medical education was encouraged while post-graduation practice was constrained. The absence of female role models in surgical specialties undermined surgical self-efficacy among female participants considering male-dominated fields.

**Conclusion:**

Career development in collectivist contexts operates through family collective agency and culturally structured participation norms that are not well captured by individualistic models. Our findings suggest SCCT may require reconceptualization to incorporate collective efficacy, honor-based outcome expectations, family approval–filtered goal formation, and cultural context as a central organizing condition. Medical education policy should recognize career “choice” as a negotiated family process rather than an exclusively individual decision.

## 1 Introduction

Career decision-making in medical education predominantly reflects Western individualistic frameworks that emphasize personal autonomy and self-directed goals (1–3). However, in collectivist cultures, families exercise collective agency over career trajectories through shared deliberation and binding decisions that supersede individual preferences (4, 5). Clinical rotations represent a critical juncture where individual training experiences intersect with the authority of family decision-making. These rotations shape specialty interests (6), professional identities (7, 8), and career trajectories (9). However, how families interpret and filter rotation-derived interests in collectivist contexts remains unexplored (10–12).

Pakistan annually educates over 15,000 medical students across 126 medical and dental colleges (9), with male and female students undergoing identical clinical rotations in years 3–5 (13). However, identical training produces divergent career outcomes: families assume post-graduation decision-making authority over specialty selection and practice settings (14, 15), with gender-specific cultural expectations constraining choices (16). Despite substantial medical training infrastructure, empirical studies examining how clinical rotations shape career decisions within collectivist family structures are limited (17, 18).

Although SCCT and CoP originate in individual-centered theoretical traditions, both frameworks include relational constructs such as social learning processes, efficacy development, and participation trajectories that allow analytical adaptation to collectivist contexts. Rather than treating culture as a peripheral moderator, this study employs these frameworks to examine how family authority, gender norms, and collective decision-making reshape core career development mechanisms, enabling theory extension rather than simple theory transfer.

Social Cognitive Career Theory (SCCT) and Communities of Practice (CoP) theory dominate career development literature; however, both originated in individualistic Western contexts (1–3, 19–21). SCCT posits that self-efficacy beliefs and outcome expectations formed during clinical training drive career goal formation (19, 20), while CoP describes how legitimate peripheral participants progress toward full participation by increasing community engagement (21, 22). These frameworks inadequately address collectivist contexts where family collective decision-making supersedes individual agency, gender-based structural barriers may prevent participation progression, and cultural honor systems mediate the relationship between training experiences and career outcomes. We use these theories as sensitizing concepts (23) to explore how career development operates when the assumptions of individual autonomy collapse. Studies from Indonesia and other collectivist cultures suggest that Western career theories have limited applicability when family influence predominates (4, 5). However, the specific mechanisms through which families filter rotation-derived interests remain unexplored.

Intersectionality theory examines how social identities interact to create unique experiences irreducible to single-axis analysis (24, 25) and provides a framework for understanding how gender operates within culturally specific systems (26, 27). In this study, intersectionality was operationalized through the simultaneous examination of gender, family hierarchy, marital expectations, socioeconomic position, and Pashtun cultural norms as interacting forces shaping clinical participation and career constraints. During rotations, female students encounter gender-based patient assignments and specialty-specific restrictions (28).

In conservative Islamic contexts, families may view medical education for women as socially acceptable and professionally respectable, particularly within gender-segregated healthcare systems (29, 30). However, long-term career participation is often constrained by cultural and religious expectations surrounding gender-concordant care and family roles (31). Families may also restrict post-graduation specialty choices as marital obligations assume priority, creating a “doctor daughter” paradox in which medical education is encouraged but professional practice is constrained (32). Male students face different pressures, including family expectations to pursue lucrative or prestigious specialties despite rotation experiences suggesting alternative fits (16). The intersection of gender within Pashtun cultural honor systems (izzat) produces distinct career barriers that cannot be understood by studying Western female medical students or Pakistani male students in isolation (33).

Mentorship and role modeling during clinical rotations significantly influence specialty interest (34, 35). Positive role models strengthen self-efficacy, and their absence diminishes specialty confidence (36, 37). However, questions remain: when female students develop an interest in surgery during rotations but encounter no female surgeon role models, how do families evaluate specialty feasibility? When male students experience emotional connection to pediatrics but cultural masculinity norms favor “technical” specialties, do rotation experiences or family expectations prevail (16)? Clinical rotation literature assumes that experiences shape individual preferences (6, 38) but minimally addresses how collectivist families interpret and override rotation-derived interests.

This study examines three questions: (RQ1) How do clinical rotation experiences influence career decision-making when family collective decision-making controls post-graduation specialty selection? Through what mechanisms do rotation-derived interests become filtered through family deliberation? (RQ2) What patterns of participation and marginalization do students experience during clinical rotations, and how do structural gender barriers influence their progression from peripheral observation to full participation? (RQ3) How do the intersections of gender and Pashtun cultural contexts shape the relationships among clinical rotation experiences, family decision-making processes, and career trajectories?

Career development theories extensively validated in Western individualistic settings have limited applicability when family collective agency supersedes individual autonomy (4, 5). Pakistan, where families exercise binding authority over specialty selection and practice settings (14, 15), offers a critical case for examining how clinical training experiences intersect with collectivist decision-making. By exploring how family systems, cultural norms, and gender shape career trajectories during clinical rotations, this study develops more inclusive theoretical models that account for the diverse cultural contexts in which most medical students worldwide are now training.

## 2 Methodology

### 2.1 Study design and theoretical framework

This qualitative study employed semi-structured interviews analyzed using reflexive thematic analysis (39) within a critical realist epistemology, recognizing both the material realities of gendered clinical structures and the socially constructed meanings students and families attach to career decisions (40). This epistemological stance acknowledges that family collective agency operates within real structural constraints (gender-based clinical barriers and specialty access restrictions) while being interpreted through culturally specific meaning systems (honor, marriageability, and breadwinner expectations). Social Cognitive Career Theory, Communities of Practice, and intersectionality were used as analytic sensitizing concepts to support theory-informed interpretation during later stages of analysis. This approach guided the inquiry while remaining attentive to patterns outside theoretical predictions. We followed the Consolidated Criteria for Reporting Qualitative Research (COREQ) reporting guidelines (41).

### 2.2 Sampling strategy and participant recruitment

We employed maximum variation purposive sampling (42) stratified by gender, discipline (medicine/dentistry), and academic year (third through fifth year with completed clinical rotations). The sample size followed information power principles (43), considering a narrow study aim, high sample specificity, strong interview dialogue quality, and an in-depth analytic strategy. These factors indicated adequate information power with 28 participants, and thematic saturation (44) was confirmed with four additional interviews.

Recruitment was conducted through institutional liaisons who distributed study information via student portals and WhatsApp groups. Students who were interested contacted the research team directly. Of the 36 approached students, four declined (11% refusal): two cited time constraints, one expressed discomfort with recording, and one declined without explanation.

Final sample: 32 participants (18 medical students: 9 females, 9 males; 14 dental students: 8 females, 6 males), aged 20–25 years (mean 22.6, SD 1.3). Alphanumeric codes indicated discipline, gender, and sequence (e.g., MF01). Full demographics are provided in Supplementary Table S1.

### 2.3 Study setting

Data were collected between August to November 2024 across four undergraduate medical and dental training institutions in Peshawar, Pakistan (two public-sector and two private-sector colleges). All follow the Pakistan Medical and Dental Council curricula, with two preclinical years followed by clinical rotations beginning in Year 3 (10). Clinical rotations include internal medicine, surgery, pediatrics, and obstetrics and gynecology (medical students) and oral surgery, prosthodontics, and community dentistry (dental students). Students learn through hierarchical apprenticeships, with senior consultants supervising postgraduate residents who oversee undergraduates.

Peshawar, the capital of Khyber Pakhtunkhwa province, has a predominantly Pashtun population, where cultural norms regarding gender roles, family authority, and arranged marriage influence professional trajectories (45, 46). Female students comprise approximately 60–70% of enrollment (47) yet face post-graduation restrictions as families direct specialty choices and practice settings (17, 32). Male students face breadwinner expectations, emotional stoicism aligned with Pashtun masculinity (*nang, mashar*), and pressure to pursue high-earning specialties. We included both medical and dental students to examine whether the collective agency of families in career decision-making operates similarly across health professions when clinical training structures differ.

### 2.4 Data collection

A gender-matched team of medical students conducted all semi-structured interviews in private campus locations during participants’ free periods, following established qualitative interviewing procedures (45). Interviews lasted 60–90 min in participants’ preferred language (Urdu, English, or Pashto).

Interview guides were developed using structured guide design principles (46) (Supplementary S2). The interview guides (Supplementary S2) explored the following: clinical rotation experiences and their influence on specialty interests; family discussions about career decisions and participants’ roles in these discussions; temporal sequences of when rotation interests formed and when family input occurred; and specific mechanisms by which families approved, modified, or rejected rotation-derived specialty interests. We explored the types of clinical tasks students performed (observation only, supervised assistance, independent performance), progression patterns across rotation duration, barriers preventing progression despite competence development, and differences in progression by gender and specialty. For female participants, we explored anticipated marriage timing, family expectations regarding post-marriage career modifications, and experiences with gender-based patient assignments and specialty restrictions. For male participants, we explored how breadwinner expectations and specialty choices were influenced by anticipated family financial responsibilities. Probes explored *izzat* (honor) pressures, *purdah* and *mahram* norms, SCCT constructs (confidence development, outcome expectations, goal adjustments), and CoP concepts (novice-to-practitioner identity shifts, community belonging).

Audio recordings were transcribed verbatim by professional transcriptionists, and bilingual team members verified transcription accuracy (47). Urdu and Pashto quotes were translated to English with back-translation verification, and culturally specific terms were retained in italics with contextual explanations. Gender-matched interviewers created reflexive memos immediately post-interview, documenting contextual observations and analytic reflections.

### 2.5 Data analysis

We followed Braun and Clarke’s six-phase reflexive thematic analysis framework (39). In the familiarization phase, all authors independently read the transcripts twice, noting initial patterns. During the initial coding, two researchers independently coded five transcripts (interviews 1, 5, 10, 15, and 20), generating 127 initial codes from the first 10 interviews. A strong consensus was achieved; disagreements (18% of coded segments) were resolved through discussion, primarily concerning distinguishing family support from family constraint, categorizing emotional stress as individual versus honor-related, and identifying participation versus marginalization. These discussions refined the code definitions and operational criteria. To examine whether CoP’s participation progression predictions held in contexts where gender barriers may prevent progression regardless of skill development, we coded the types of clinical tasks performed, progression patterns across rotation duration, barriers preventing progression despite competence, and differences in progression by gender and specialty.

During theme generation, the codes were collated into 12 candidate themes through iterative mapping. We prioritized semantic themes (explicitly stated experiences) over latent themes to maintain descriptive rigor in collectivist contexts, where Western psychological constructs may not transfer. In the theme review phase, all the authors reviewed the themes against the coded extracts and full transcripts. Five candidate themes were collapsed owing to conceptual overlap, and two were split to capture distinct gender patterns. Final structure: five themes and 14 subthemes. During the defining and naming phase, themes were defined with clear boundaries, and subtheme labels were vetted with two participants for cultural resonance (member reflections, not validation). In the final reporting phase, an analytic narrative was constructed to balance the descriptive data presentation (Results) with the theoretical interpretation (Discussion). Quotes were selected based on typicality or illuminative power.

The analysis employed abductive reasoning (48), moving iteratively between empirical data and theoretical constructs. Although the interview questions incorporated theoretical constructs, we remained open to patterns outside these frameworks. The initial coding was open and exploratory, and theoretical frameworks informed later interpretations. Gender-stratified analysis compared female and male experiences. We actively sought negative cases challenging patterns under development; three male students (MM02, MM05, MM09) described peer emotional support networks, contrasting with the dominant emotional suppression pattern in the other interviews. The final codebook contained 78 codes in 12 categories (Supplementary S3). NVivo 12 software was used for data management and coding organization (49). Sample-coded excerpts demonstrating each theme are presented in Supplementary S4.

### 2.6 Quality assurance

We applied Lincoln and Guba’s trustworthiness framework (50). Credibility was supported through prolonged engagement and member checking. Member checking with 26 of 32 participants (81% response rate) who reviewed preliminary theme summaries 2 to 4 weeks post-interview (23 affirmed accuracy, three suggested nuances incorporated into final themes; Supplementary file S5.2), and data source triangulation across four institutions, both professions, and multiple rotation types.

Dependability was addressed through audit trails documenting coding decisions, theme development, consensus discussions and reflexive memos (Supplementary S5). Confirmability was established through reflexive journals documenting the researcher’s assumptions, emotional responses, and interpretation development (Supplementary S5.1). Transferability was enhanced through thick description (51). Peer debriefing was conducted with two external medical education researchers who challenged the interpretations, particularly regarding whether family influence operated as coercive control or collaborative negotiation (Supplementary S5.4). We completed the COREQ 32-item checklist (41) (Supplementary Section S7).

### 2.7 Ethical considerations

This qualitative study followed the ethical principles of the Declaration of Helsinki and was approved by the Prime Foundation Institutional Review Board (protocol code: Prime/ERC/2025-48, approved on 20 July 2024). Participants provided written informed consent after reviewing information sheets explaining the study’s purpose, procedures, risks, benefits, and their right to withdraw.

Pseudonyms followed the coding system described in Section 2.3. No identifying information was included in the publications. Transcripts and audio files are stored in password-protected encrypted servers accessible only to the research team members.

## 3 Results

**Figure 1:**
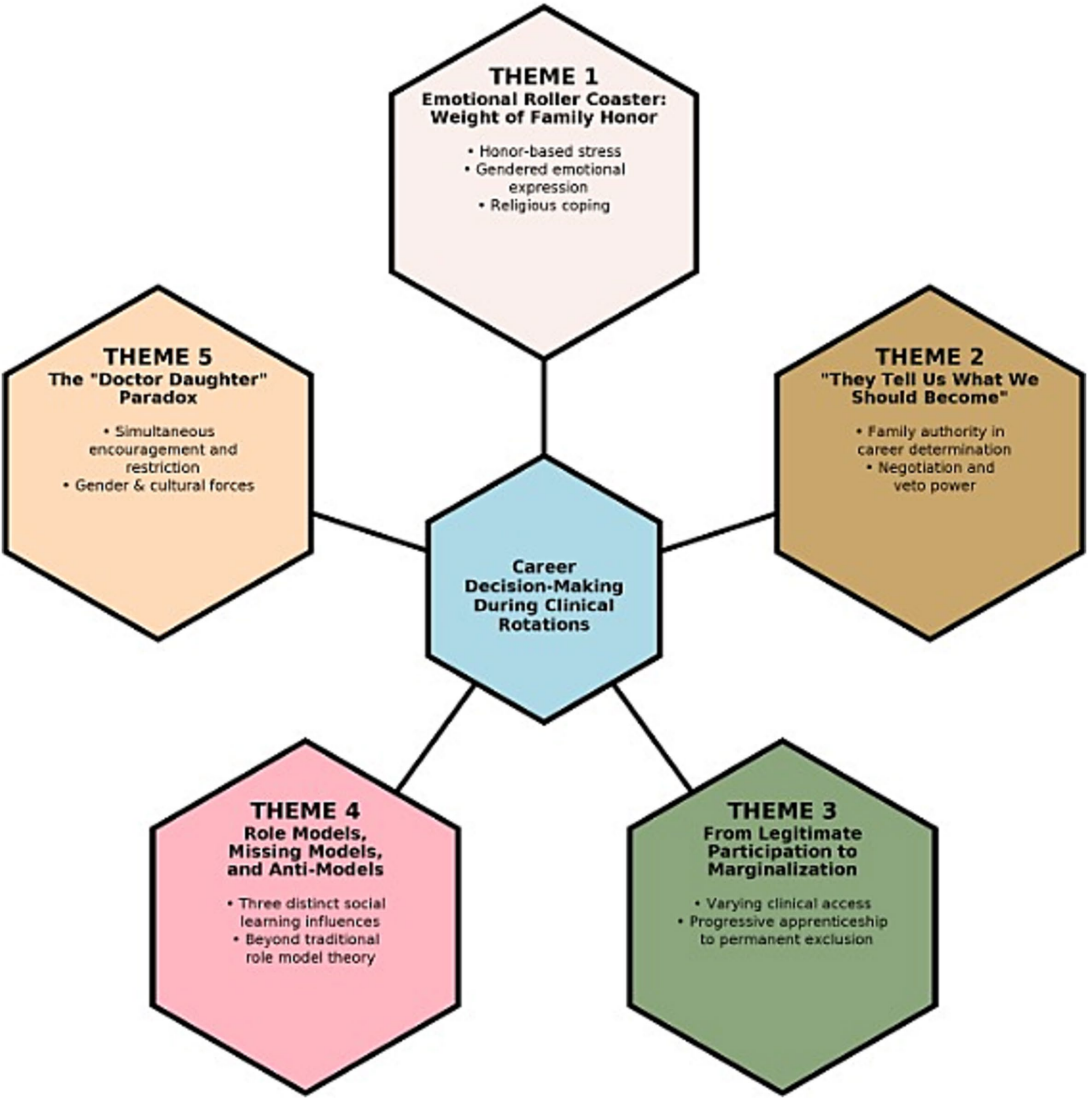
Thematic map: intersecting influences on career decision-making during clinical rotations. Figure showing the central hexagon representing career decision-making processes, surrounded by five interconnected themes in a balanced pentagon arrangement. Theme 1 (Emotional Roller Coaster) examines honor-based stress, gendered emotional-expression norms, and religious coping strategies. Theme 2 (“They Tell Us What We Should Become”) describes the family’s collective agency in career determination through negotiation and veto power. Theme 3 (From Legitimate Participation to Marginalization) documents varying clinical access from progressive apprenticeship to permanent structural exclusion. Theme 4 (Role Models, Missing Models, and Anti-Models) identified three distinct social learning influences shaping specialty self-efficacy. Theme 5 (The “Doctor Daughter” Paradox) reveals how female students experience simultaneous encouragement for medical education and restrictions on career possibilities. The pentagon arrangement illustrates the equal importance and interconnected nature of themes, with gender and the Pashtun cultural context operating as cross-cutting dimensions shaping all five themes.

Five major themes emerged from the reflexive thematic analysis (Figure 1; coding framework in Supplementary Section S3). Participant codes followed the format: MF (Medical Female), MM (Medical Male), DF (Dental Female), and DM (Dental Male), followed by sequential numbers (demographics Supplementary Table S1). Interviews 29–32, conducted after the initial thematic saturation at interview 28, confirmed thematic stability without introducing substantively new dimensions.

Students described intense emotional shifts during clinical encounters, such as excitement transitioning to anxiety and confidence collapsing into inadequacy, often within a single rotation. Clinical stress extends beyond individual performance evaluation to encompass family reputation. MM04 (fifth year) explained: *“When you make a mistake in front of patients, it is not just your failure. You are representing your family’s name (izzat). That pressure is always there—every patient interaction carries your family’s honor.”* This honor-based dimension of clinical stress was reported across medical and dental cohorts, and across genders.

Emotional responses followed gendered patterns shaped by Pashtun cultural codes. Male students described cultural prohibitions against vulnerability rooted in *nang* (honor requiring courage, independence, and emotional stoicism). MM08 (fifth year) reflected: *“Men do not show weakness, especially not in clinical settings where patients and seniors are watching. Even when I was terrified during my first surgery rotation—hands shaking, nauseated–I had to appear confident. If I had shown fear, it would reflect badly on my manhood (nang) and my family’s reputation. Pashtun men are supposed to be strong.”* Majority of male participants described pressure to maintain emotional composure even in private peer interactions, with several explicitly linking this expectation to Pashtun masculinity norms.

A small subset of male medical students (MM02, MM05, MM09) reported exceptions within small, long-standing friendship circles (3–4 students together since preclinical years). MM05 (fourth year) explained: *“My close friends and I talk honestly about struggling. We pray together after difficult cases, make dua for patients who died, and ask Allah for strength. Perhaps because we have been together since the first year, there is trust that what we share will not leave the group. But it’s not like Western ‘therapy talk’, we frame struggles through faith, not psychology.”* These exceptions involved religious legitimization of emotional expression (e.g., collective prayer, seeking divine assistance) rather than secular psychological disclosure.

Female students accessed familial emotional support more readily through their mothers, sisters, and female cousins. However, they also described systematic dismissal of clinical stress as evidence of unsuitability for more demanding specialties. MF07 (fourth year) recounted: *“When I told my surgery senior I was stressed managing multiple post-operative patients, he said, ‘Maybe surgery is not for girls who cannot handle pressure. You should think about something easier like pediatrics or dermatology.’ However, I knew that the male students on my team were equally stressed; one of them told me privately that he was not sleeping. They could not admit it publicly. So identical stress responses got interpreted completely differently based on gender.”* This pattern was described frequently by female participants, particularly in surgery, emergency medicine, and orthopedics rotations.

Religious coping mechanisms, such as prayer (salah), Quranic recitation, remembrance of Allah (dhikr), and seeking blessings through family elders’ prayers (dua), served as primary stress management strategies for both genders. MF09 (fifth year) reflected: *“My mother’s prayers give me strength. When she tells me that she is praying during my exams or difficult rotations, that knowledge carries me through. I also recite Ayat al-Kursi before entering the hospital each morning, asking Allah for knowledge to help patients and strength to handle what I’ll see.”* Religious coping was a dominant response across interviews. Most participants mentioned religious practices as their primary coping mechanism. No participant described using formal psychological counseling services. Three dental students (DF02, DF06, DM03) stated they would not use counseling services, even if available, due to mental health stigma, and preferred guidance from religious scholars or family elders. DF02 explained: *“Mental health services are for people who are actually sick. For normal stress, we turn to faith and family for support. That’s our culture.”*

Female students whose stress was dismissed as “specialty unsuitability” subsequently avoided those fields despite having sustained clinical interest. Male students’ emotional suppression prevented them from seeking mentorship when they were struggling. Students who relied on their parents’ prayers also described feeling obligated to honor their parents’ specialty preferences. MM06 stated: *“My mother prays tahajjud [pre-dawn prayer] for my success. How can I ignore her wishes regarding my specialty? Her prayers are why I’m succeeding; rejecting her guidance would be ungrateful and dishonor her sacrifice.”*

Career decisions unfolded through family deliberation processes rather than individual determinations. Students described ongoing discussions involving fathers (typically financial authorities), mothers (often advocating for children’s expressed preferences), extended family elders (*mashar,* whose opinions carry significant weight), and older siblings (providing career insights). The family collective functioned as the decision-making unit, with students’ voices representing one input among several rather than a determinative authority.

Family veto power was explicitly exercised when personal interests conflicted with family economic needs or prestige expectations. MM06 (fourth year) stated: *“I genuinely wanted psychiatry after my rotation. I found the diagnostic complexity fascinating and the therapeutic relationship meaningful. However, my father outright refused when I mentioned it. He said, ‘I did not invest hundreds of thousands of rupees in your medical degree for you to become a psychiatrist. Nobody respects that field here, and the income is minimal. You’ll do medicine or surgery – something with proper respect and earning potential.’”* Economic framing was a dominant narrative among male participants, with several stating that they would pursue specialties they did not prefer to fulfill family financial obligations.

MM09 (fifth year) elaborated on breadwinner expectations: *“My family spent everything on my education. My father took loans, and my mother sold jewelry for my test preparation fees. My younger brother and sister are waiting for me to start earning so that they can attend university. How can I selfishly choose community medicine, which I love, when it barely pays enough for one person? I have to choose a high-income specialty, probably radiology or cardiology, even though I find them isolating. My personal interest is a luxury my family’s economic situation doesn’t permit.”* Many male participants described similar pressures, with specialty trajectories funneling toward radiology, cardiology, surgical subspecialties, and anesthesiology regardless of rotation-derived interests.

Female students consistently identified marriage specifically the future husband’s family’s expectations as the primary career determinant superseding current plans. MF03 (fifth year) explained: *“Everything depends on who I marry and what his family expects. If my future husband and in-laws support my career, I can pursue surgery, which I genuinely enjoy. If they expect me to prioritize my home and children, I will need something with controllable hours, such as family medicine or dermatology, or I may even stop practicing. I can’t make concrete plans until I know their expectations, which I won’t know until after marriage arrangements are made.”* Female participants consistently described this form of contingent planning, regardless of academic performance or strength of specialty interest.

DF01 (third year) elaborated: *“After marriage, my in-laws’ preferences will matter more than my parents’ or mine. That is our culture; when you marry, your primary loyalty shifts to your husband’s family. If they want me to practice part-time, only treat female patients, or stop working to focus on children, that will be my reality. So, I am already thinking strategically about specialties where part-time practice is feasible and where I could stop for several years and resume later.”* A substantial subgroup of female participants explicitly anticipated post-marriage practice restrictions regardless of current aptitude, viewing career planning as necessarily provisional pending marital arrangements typically occurring one to 3 years post-graduation.

Family involvement was not uniformly restrictive. MM03 (third year) described: *“I had planned to study engineering after secondary school. I was strong in mathematics, and I believed that engineering would lead to good jobs. However, my parents, especially my father, who works in healthcare administration, convinced me to pursue medicine instead. They said, ‘You have the ability, medicine provides more security, and you can serve people.’ Honestly, I was not interested during the first two preclinical years. However, after starting clinical rotations and treating patients, and witnessing their relief when symptoms improved, I genuinely started to like it. I cannot imagine doing anything else. My parents saw potential I didn’t recognize myself.”*

DF07 (second year) described collaborative family dynamics: *“My parents regularly ask what I’m enjoying in rotations, what suits my personality, what lifestyle I envision. We discuss the pros and cons of different specialties together; they share insights from their friends who are dentists, and I share what I am learning. But ultimately, they say it is my decision, and they will support whatever I choose as long as I choose thoughtfully. Their trust and support makes me feel confident I’ll make good decisions.”* A minority of participants described such collaborative family dynamics, in which parents provided guidance and financial support while ultimately deferring specialty decisions to students.

Patterns varied according to family socioeconomic context, student gender, and birth order. Economically stable families granted students greater autonomy, whereas families facing economic precarity exercised tighter control. Sons generally received more decision-making autonomy than daughters. Eldest children, particularly eldest sons, faced the greatest pressure toward high-income specialties to support younger siblings’ education, while younger siblings in families with already established earners experienced substantially greater flexibility.

Clinical learning experiences varied dramatically along a continuum from full legitimate peripheral participation (progressive skill building toward independent practice) to permanent marginalization (exclusion from meaningful learning regardless of competence development).

Some rotations exemplified an ideal progressive apprenticeship. MM07 (fifth year) described his internal medicine rotation: *“The consultant had a clear teaching philosophy, he believed in graduated responsibility. In the first week, we watched him take histories and examine patients while he explained his clinical reasoning aloud. In the second week, we took histories under direct supervision with immediate feedback. In the third and fourth weeks, we examined patients independently and presented cases during morning rounds, with him asking probing questions to develop our diagnostic thinking. By the end of the rotation, I felt like a real doctor, not just a student observer. That experience is why I’m choosing internal medicine.”* Some participants described at least one rotation following this progressive trajectory.

Other rotations relegated students to passive observation without conceptual understanding or team integration. MF02 (fourth year) described herself as *“furniture in the corner which is present in the room but serving no function, acknowledged by nobody.”* MF04 (fourth year) described her surgery rotation: *“We just stood silently in the operating room for hours, trying not to faint or move. No one explained what they were doing or why. When we asked questions, they would say, ‘just watch and learn,’ but watching without context teaches nothing. I could not see the surgical field from where we stood, hear the quiet discussions between the surgeon and anesthesiologist, or understand the decision-making. I learned more from YouTube videos than from actual OR time.”* Experiences of marginalization were described by reported frequently, with surgery rotations disproportionately referenced.

Structural barriers further limited learning access. Students described large rotation cohorts (15–20 students per team) competing for limited hands-on opportunities, faculty time pressures prioritizing clinical efficiency over teaching in high-volume public hospitals, short rotation durations (3–4 weeks) limiting relationship development with supervisors, and hierarchical competition in which postgraduate residents controlled undergraduates’ access to learning opportunities while prioritizing their own training needs.

Gender-structured participation emerged through explicit institutional policies and informal cultural norms. Female students described systematic exclusion from male patient examinations, even when patients expressed consent. MF05 (fourth year) stated: *“There was a young male patient with a hernia that needed examination. He explicitly stated that he was comfortable with female medical students examining him and that he wanted to help us learn. However, the senior doctor said, ‘It is not appropriate for female students to examine male patients in these areas. It’s better you learn this from textbooks.’ But how will we learn about male genitourinary anatomy, hernias, and testicular examinations from textbooks? These are clinical skills requiring hands-on practice.”* Across interviews, female participants reported experiencing gender-based exclusion from male patient examinations in medicine and surgery rotations, with many describing concerns that these gaps would translate into examination and competency disadvantages.

In Peshawar’s medical colleges, night shifts and emergency duty rotations remained largely inaccessible to female undergraduate students due to institutional safety concerns and family propriety expectations rooted in purdah (gender segregation norms). MF06 (fourth year) explained: *“Night duty is when you really learn emergency management, acute presentations, rapid decision-making, working with limited resources, and handling crises independently because senior faculty are not immediately present. That is where you develop confidence. However, female students were not allowed for safety reasons. This contrasts with other parts of Pakistan. I know female medical students in Karachi and Lahore do night shifts during medical school, but here in Peshawar, we’ll only get that experience during house job after graduation.”* Female participants described night duties as largely inaccessible, whereas male participants more commonly described access.

Male students faced parallel gender-based barriers in obstetrics and gynecology, where patient refusals and institutional policies severely limited exposure. MM09 (fifth year) explained: *“Many female patients refuse to be examined in front of male medical students, sometimes for religious modesty reasons, sometimes because having male students present during labor and delivery feels invasive, and sometimes because their husbands refuse on their behalf. Therefore, our exposure is extremely limited. My entire OB/GYN knowledge is theoretical. I completed the six-week rotation without performing a single pelvic examination or attending a delivery. Everything I ‘learned’ came from textbooks and watching from distances where I couldn’t see or understand what was happening.”* Most male medical students described completing OB/GYN rotations with minimal hands-on participation.

Repeated exclusion shaped students’ self-concepts and specialty trajectories. MF08 (fourth year) reflected: *“When you are repeatedly told ‘you cannot do this procedure because you are female’ or ‘step back, let the male students try first,’ you start internalizing that certain fields are not for you. Not because you objectively lack ability, I have excellent grades and good hand skills but because you lack the opportunity to develop that ability through practice. By the time you reach your final year and need to make specialty decisions, you have already internalized these limitations. The barriers become part of your self-concept.”* Many female participants described diminishing confidence in procedurally intensive specialties despite strong academic performance.

Dental students reported similar gendered participation patterns. DF04 (fourth year) stated: *“Male patients frequently refuse female dental students for tooth extractions, saying it is not appropriate for women to be that close to their faces or that they doubt our physical strength. Therefore, our male classmates accumulate far more oral surgery experience. By graduation, they might have performed 50–60 extractions; we might have performed 15–20.”* DM05 (fourth year) described a contrasting experience in prosthodontics: *“The senior prosthodontist gave us graduated responsibility by first observing crown preparations, then doing them under supervision, and finally managing complete cases independently. I felt truly competent by the end.”* DM02 (second year) added: *“Female patients sometimes refuse male dental students, especially for anterior tooth work where we are very close to their faces. So, both genders get partial training, just with different gaps. We learn more from male patients, they learn more from female patients.”* DF06 (fourth year) reflected: *“I was rarely allowed to perform extractions on male patients. The consultant would say, ‘Let the boys handle this one, it requires force.’ But I never got the chance to develop that force or technique because I was excluded from practicing.”*

### 3.1 Theme 4: role models, missing models, and anti-models

Three distinct social learning influences emerged: (1) positive role models whose attributes inspired emulation; (2) missing models whose absence undermined specialty feasibility beliefs; and (3) anti-models whose behaviors or lifestyles actively deterred specialty interest despite positive clinical experiences.

#### 3.1.1 Positive role models

MM02 (fourth year) described an internal medicine consultant: *“He was brilliant in making differential diagnoses. He could identify obscure conditions from subtle clinical signs that others missed, but he was also incredibly kind to patients. He sat on patients’ beds, held their hands when delivering bad news, and spent extra time explaining treatment plans in simple Urdu or Pashto. More importantly for us students, he took time to teach his clinical reasoning process. He asked, ‘What made you think of that diagnosis? Walk me through your reasoning,’ then showed us where our thinking was sound and where we made logical leaps. He treated us as junior colleagues, not as burdens. I want to be that kind of doctor, excellent clinically but also humanistic and teaching focused.”*

Many participants described encountering at least one positive role model during their rotations.

DM01 (third year) described a prosthodontics consultant as follows: *“She was meticulous with crown preparations, teaching us proper margin placement, explaining occlusal considerations, and supervising our lab work closely. However, she also made time to discuss practice management, patient communication strategies, and building referral networks. She showed us that prosthodontics is not just a technical skill; it is about building relationships with patients and labs. That’s why I’m choosing prosthodontics.”*

#### 3.1.2 Missing models

MF08 (fifth year) explained: *“I was genuinely interested in orthopedics. I loved the biomechanical problem-solving, the immediate tangible results of surgical intervention, and the active patient population. However, there is not a single female orthopedic surgeon in our entire hospital system or any hospital my family knows in Peshawar. When I try to imagine myself in that career, I cannot picture how I would manage it as a woman. How would I handle the physical demands of pregnancy? How can I balance orthopedic call schedules with family expectations after marriage? How would I navigate being the only woman in a hyper-masculine surgical subspecialty? These questions have no answers because no woman has done it here. The absence itself is a message: ‘women don’t do this.’”*

Similar concerns were expressed by female participants regarding surgery, orthopedics, urology, and interventional cardiology.

When female students expressed interest in specialties lacking female practitioners, families frequently used this absence to discourage their choices. MF09 (fifth year) stated: *“When I mentioned neurosurgery to my family, they said, ‘No woman has succeeded in neurosurgery in Peshawar, so it must not be possible for women. Choose something realistic.’”*

DF05 (fourth year) reflected: *“There are very few female oral and maxillofacial surgeons in our city. When I expressed interest after my oral surgery rotation, my family asked, ‘Who will you learn from? Where are the female role models?’ The absence made my interest seem impractical, even though I had strong surgical skills.”*

MM04 (fifth year) reflected: *“There are very few male nurses and very few male pediatricians in our hospital. Pediatrics is seen as ‘motherly work’. Nurturing, gentle, emotionally expressive, which conflicts with Pashtun masculine ideals. Even though I really enjoyed my pediatrics rotation, I felt like I’d be an outsider, constantly questioned about why I chose a ‘female specialty.’ Where are the male role models who could show that men can be excellent pediatricians without threatening their manhood?”*

#### 3.1.3 Anti-models

Clinicians whose characteristics actively deterred specialty interest shaped career decisions. Three types emerged.

#### 3.1.4 Lifestyle anti-models

MF01 (fourth year) explained: *“One of our senior surgery consultants is clinically brilliant and I admired his technical skill and decision-making. But I watched his life over six weeks and thought, ‘I don’t want that existence.’ He was called away from family dinners three times during my rotation for an emergency surgery. His daughter came to the hospital once, crying because he had missed her school event again. He appeared to be perpetually exhausted. I want to be a good doctor, but I also want a life outside medicine that is time for family, hobbies, and spirituality. His life was only surgery.”*

Several participants similarly cited work–life imbalance as a reason for abandoning initially attractive specialties, particularly surgery and emergency medicine.

DF08 (fourth year) described a comparable experience in oral surgery: *“Our oral surgery consultant was always exhausted, managing emergencies at all hours. His wife would call during the clinic and ask when he would be home. I thought, ‘I love the surgical aspect, but do I want that lifestyle?’ It made me reconsider.”*

#### 3.1.5 Character anti-models

MM08 (fourth year) stated: *“I genuinely enjoyed the clinical work in surgery, the problem-solving, the immediate results, the technical challenge. However, the culture was toxic. I watched senior residents scream at nurses over minor issues, consultants publicly humiliate students who asked, ‘stupid questions,’ everyone tolerating it as ‘that’s just how surgery is.’ I realized that I did not want to become that kind of person, and I was afraid that if I trained in that environment for five years, I would absorb those values. So, I’m choosing medicine instead, even though surgery interested me more clinically.”*

Several participants described rejecting specialties primarily due to toxic workplace cultures rather than lack of clinical interest.

#### 3.1.6 Sacrifice anti-models

MF02 (fourth year) reflected: *“One of our senior female surgeons is excellent clinically and probably the best technical surgeon in the department. However, she is unmarried at 40, and I know her family constantly pressures her about this. Everyone whispers she ‘chose career over family.’ She appears lonely despite her professional success. I admire her professionalism. She is proof that women can succeed in surgery, but I do not want that struggle or social judgment. I want both career and family, which means I probably can’t choose surgery.”*

Several female participants described successful senior female doctors as cautionary examples rather than aspirational role models.

### 3.2 Theme 5: the “doctor daughter” paradox*—*intersecting gender and Pashtun culture

Female students described a consistent paradox in which families strongly encouraged medical education while simultaneously restricting post-graduation career possibilities. DF01 (third year) explained: *“My father was so proud when I got into dental college. He tells everyone about his ‘doctor daughter’ (ḍākṭar beṭī) with obvious pride; it brings honor to our family. He paid for expensive test preparation courses without any hesitation. However, in the same conversation, he also says, ‘After marriage, your in-laws’ expectations come first. If they want you home raising children, you’ll respect that.’ Therefore, he invested lakhs of rupees, knowing that I might never use my degree fully or might practice only part-time. It’s contradictory but somehow makes sense within our cultural logic.”*

Across interviews, female participants consistently described this tension and offered multiple cultural explanations. Some emphasized community service obligations, particularly the need for female physicians to treat female patients within purdah-restricted clinical contexts. Others highlighted family prestige (izzat), where educated daughters enhanced social standing and marriage prospects in competitive matrimonial markets. Additional participants framed medical education as economic insurance against marital instability, widowhood, or spousal financial insecurity. Despite educational encouragement, families simultaneously emphasized post-marriage domestic responsibilities, including childcare, household management, and obligations toward in-laws, positioning professional careers as secondary or conditional.

Interpretations varied by family background. Students from more urban or educationally privileged households emphasized prestige and public service narratives. Those from economically vulnerable families highlighted financial security motives. Students from particularly conservative households emphasized domestic role primacy as the central justification.

The most tangible consequence of this paradox was anticipatory specialty screening. Female students reported proactively eliminating career options based on predicted post-marriage incompatibility rather than present aptitude or interest. MF03 (fifth year) stated: *“I genuinely love surgery. My surgery rotation was the best experience of medical school, the adrenaline of the OR, the immediate problem-solving, the visible results, and the team coordination under pressure. I was good at it; my supervising resident said I had ‘excellent surgical hands.’ However, everyone; my mother, senior female doctors, and married female friends of the family tells me that surgery is incompatible with married life in our culture. They say, ‘Choose something family-friendly that you can do part-time or resume after children.’ Therefore, I am preparing to let that interest go, even though it hurts deeply. I’m applying to family medicine positions instead, not because I prefer it, but because it’s what is possible for women who want families, which is non-negotiable in our culture.”*

Participants described repeatedly narrowing specialty aspirations toward fields perceived as compatible with marriage and domestic expectations, including dermatology, pediatrics, family medicine, and radiology, while procedurally intensive specialties such as surgery, orthopedics, emergency medicine, and obstetrics were framed as socially incompatible regardless of performance or interest. MF07 (fourth year) summarized this constraint: *“Interest becomes secondary to feasibility. We learn early to separate what we want from what we can have.”*

Similar patterns emerged among dental students. DF03 (third year) explained: *“Oral and maxillofacial surgery requires long hours, emergency calls, and hospital rounds. My family says that it is impossible after marriage. They’re steering me toward pediatric dentistry or orthodontics. These specialties are with predictable hours and private practice settings where I can control my schedule.”* DF09 (fifth year) added: *“I loved prosthodontics during my rotation; the precision, the artistry, the patient transformations. However, it requires long appointments and complex laboratory coordination. My mother says, ‘Choose something simpler like general practice where you can see patients for 30 minutes and go home.’ So, I’m abandoning prosthodontics before I even try.”*

Male students experienced parallel constraints grounded in Pashtun masculinity norms emphasizing breadwinner status, prestige hierarchies, and emotional restraint. MM06 (fourth year) stated: *“Our society expects men to be high earners capable of supporting extended family, wife, children, parents, and younger siblings. When I mentioned interest in family medicine after my rotation, people asked, ‘Why waste your medical degree?’ My uncle said, ‘You will never be able to afford to marry on a family medicine salary.’ My interest, which I found intellectually meaningful and humanistically satisfying is treated as naïve.”*

Male participants described repeated pressure to avoid specialties perceived as low income or low prestige, including psychiatry, community medicine, family medicine, and public health, even when rotation experiences generated sustained interest. MM01 (fourth year) reflected: *“My genuine interest in psychiatry was dismissed as impractical and slightly shameful. My father said, ‘Choose something that makes money and commands respect.’ So personal interest becomes a luxury.”* DM04 (fourth year) described similar pressure in dentistry: *“Community dentistry aligned with my values, but my father said, ‘Nobody makes money there. You need orthodontics or implants.’”*

Both male and female students thus experienced constrained career agency, operating through distinct but parallel cultural logics: marriage-primacy expectations limiting women’s career intensity, and breadwinner-primacy expectations limiting men’s specialty autonomy.

Participants emphasized that in Peshawar these dynamics were rarely implicit. Families explicitly advised daughters to select “family-friendly” specialties in anticipation of marriage. Faculty members openly discouraged women from entering certain fields. Respect for elders (*mashar*) was framed as a moral obligation. MF06 (fourth year) stated: *“It’s not hidden at all. My mother openly tells me, ‘Do not choose surgery because your future husband might not allow call schedules.’ There’s no subtext — it’s explicit.”*

What remained less visible were additional institutional dynamics: financial incentives conflicting with publicly stated service ideals; patronage networks (wasta) shaping residency access and career advancement; and normalized hierarchical mistreatment framed as cultural discipline or professional rigor.

Several participants explicitly articulated the intersection of gender, Pashtun cultural norms, family hierarchy, economic class, and religious interpretation as inseparable influences on their career trajectories. MF08 (fifth year) summarized: *“You cannot separate gender from culture, family structure, class, and religion here. Being a female doctor in Peshawar is different from Karachi or London. Western feminist ideas don’t map neatly onto our realities. Our constraints are different but so are our sources of strength: family support, religious purpose, and community respect.”*

## 4 Discussion

This study examined how clinical rotations influence career decision-making among medical and dental students in Peshawar, Pakistan, through 32 in-depth interviews analyzed using reflexive thematic analysis. Five interrelated themes suggested that clinical learning operates within collectivist cultural frameworks that are not fully captured by existing Western-derived career development theories, thereby challenging the individualistic assumptions embedded in the Social Cognitive Career Theory and Communities of Practice frameworks. Rather than rejecting these frameworks, our findings indicate how SCCT and CoP may require structural reinterpretation when learning and career development are mediated by family authority, cultural honor systems, and gendered participation norms.

### 4.1 Collective honor and emotional experience

Clinical stress encompassed family honor (izzat) for 87.5% of participants, a dimension largely absent from Western medical education literature emphasizing individual burnout and resilience (52–54). Male students suppressed emotional responses to avoid violating Pashtun masculinity norms (*nang*), while female students’ stress expressions were dismissed as specialty unsuitability despite identical male stress being normalized. Three male students (MM02, MM05, MM09) reported exceptions within friendship circles where vulnerability became acceptable when framed through religious support-seeking, suggesting that emotional norms are micro-contextually variable and relationally mediated (55, 56). Notably, the salience of religion as a coping frame aligns with evidence from other Arab/Muslim-background contexts showing that distress is often interpreted and managed through culturally embedded stressors and supports (56). Interventions addressing medical students’ mental health in similar contexts may need to engage religious frameworks rather than imposing secular psychological models that may carry cultural stigma.

### 4.2 Career as family negotiation

Career decisions operated through “collective agency” with families functioning as primary decision-making units rather than influential advisors. The majority of male students reported family veto power when personal interests conflicted with economic obligations, while all female participants identified marriage (specifically, future in-laws’ expectations) as the primary career determinant. We distinguish “contingent planning” (career decisions organized around anticipated but uncertain future events controlled by others) from Western “contingency planning” (developing backup options while maintaining individual agency). Contingent planning reflects that career trajectories are negotiated through socially regulated participation and belonging, rather than determined solely by individual preference (57). Family involvement ranged from limited collaborative negotiation to dominant authoritative veto, distinguished by socioeconomic security, gender, and birth order. These findings extend the literature on collectivist career development (20, 21) by specifying the mechanisms through which family influences operate. Comparable patterns of family-mediated specialty prioritization and life-course planning have been observed in other settings, including high-income contexts where family planning considerations shape specialty selection (58), highlighting that family influence is globally relevant but structurally intensified within collectivist systems.

### 4.3 Permanent peripherality in gendered learning

Clinical learning varied from progressive apprenticeship experiences to patterns of persistent marginalization, challenging Communities of Practice theory’s assumption that learners naturally progress from legitimate peripheral participation toward fuller participation as competence develops (17, 18, 59). All female participants reported systematic exclusion from male patient examinations; most female medical students were excluded from night shifts; and nearly all male medical students faced severe obstetrics and gynecology restrictions. These findings extend prior research (12, 26) by demonstrating how cultural gender norms produce systematically different training experiences despite identical formal curricula.

We distinguish three peripherality types: (1) developmental peripherality (CoP’s original concept; competence enables fuller participation), (2) structural peripherality (permanent marginalization where social identities restrict access regardless of competence), and (3) contested peripherality (ambiguous spaces where exceptional students partially negotiate identity-based barriers). This typology reframes CoP trajectories by demonstrating that participation pathways are not merely pedagogical processes but are structurally regulated by cultural legitimacy and institutional gatekeeping in collectivist clinical environments. This extends prior critiques of CoP theory in identity-stratified learning spaces (60, 61). Cumulative marginalization produced widespread self-efficacy erosion among female students, driven not by performance deficits but by systematic denial of opportunities to develop procedural competence. These patterns subsequently influence family career negotiations and anticipatory specialty screening, creating interconnected feedback loops that progressively narrow career possibilities.

### 4.4 Role model influences

Three social learning influences emerged: positive role models (reported by most participants), missing models (undermining self-efficacy for many female participants in male-dominated specialties), and anti-models (deterring through commonly reported lifestyle imbalance, frequently reported toxic workplace behavior, and career–family sacrifice concerns among many female participants). The “absence-as-message” phenomenon proved powerful (62): female students interested in surgery encountered no female practitioners, interpreting structural absence as evidence that women cannot succeed. Families used this absence to legitimize career redirection. This extends the role modeling literature (34–37) by demonstrating that absence itself functions as a hidden curriculum, reinforcing structural exclusion cycles. Addressing missing-model effects therefore requires institutional faculty diversification rather than individual-level confidence interventions. Anti-models shaped decisions as powerfully as positive models through aversive learning, suggesting that role modeling effects depend on perceived life trajectories rather than isolated professional behaviors. Comparable narratives of identity conflict and exclusion have been documented among female surgeons in other collectivist contexts (63), reinforcing the structural nature of these deterrents.

### 4.5 The “doctor daughter” paradox

All female participants experienced simultaneous encouragement for medical education and restriction of post-graduation practice, revealing intersecting forces: female doctors fulfill community needs, educated daughters enhance family prestige and marriage prospects, medical degrees provide economic insurance against marital instability, yet domestic responsibilities supersede careers post-marriage (29, 30). Consequently, most female participants preemptively eliminated specialties based on anticipated marital constraints. Surgery, orthopedics, emergency medicine, and obstetrics were perceived as incompatible, whereas dermatology, pediatrics, family medicine, and radiology were labeled “family friendly.” Over half of male participants faced parallel constraints through breadwinner expectations that discouraged lower-income specialties. These findings add nuance to gender discrimination narratives by demonstrating that both genders experience constraint through culturally differentiated logics. Similar gendered career filtering has been reported across regional professional training contexts (63), supporting the transferability of this phenomenon beyond Pakistan.

### 4.6 Theoretical contributions and extensions

#### 4.6.1 Extended SCCT framework for collectivist contexts

Standard Social Cognitive Career Theory positions self-efficacy as individually constructed, driving career goal formation (15, 16). Our data suggest four extensions.

First, collective efficacy operates in conjunction with individual self-efficacy. Students evaluated their career capabilities through both personal performance and family collective capacity to support training. When female students were excluded from surgical rotations, it undermined both personal and family beliefs about surgical feasibility. In collectivist contexts, self-efficacy operates simultaneously at multiple levels, with family level efficacy beliefs sometimes superseding individual beliefs (64, 65).

Second, family honor (izzat) shapes the outcome expectations. Standard SCCT focuses on personal satisfaction, income, work-life balance, and social status (15, 16). Our participants also weighed whether specialties would bring family honor or shame, with some culturally coded as incompatible with gender roles. Outcome expectations prominently featured economic security for the extended family, including obligations to fund younger siblings’ education, dimensions less emphasized in individualistic SCCT applications (20).

Third, career goals operate through the family approval filter. Standard SCCT posits relatively direct pathways from self-efficacy to goal formation (15, 16). Our data indicate that in collectivist contexts, personal interests and self-efficacy beliefs must pass through family approval filters before crystallizing into goals that can be pursued. Students often abandoned their interests despite strong self-efficacy when their families intervened. Self-efficacy and outcome expectations appeared to shape interest formation more consistently than goal formation; goals were more likely to be pursued when family collective efficacy and approval aligned with individual interests, which may help explain why female students’ surgical self-efficacy did not predict surgical goal formation (66). Recent multinational SCCT-based work similarly demonstrates that contextual social forces reshape core SCCT pathways (67), supporting the need for structural adaptation rather than superficial cultural adjustment.

Fourth, cultural context functions as a central organizing framework rather than a peripheral moderator. Norms around gender (*purdah*), elder authority (*mashar*), and honor (*izzat*) define the field of perceived career possibilities. This reconceptualization positions culture not as background influence but as the primary architecture through which SCCT processes operate in collectivist settings.

**Figure 2:**
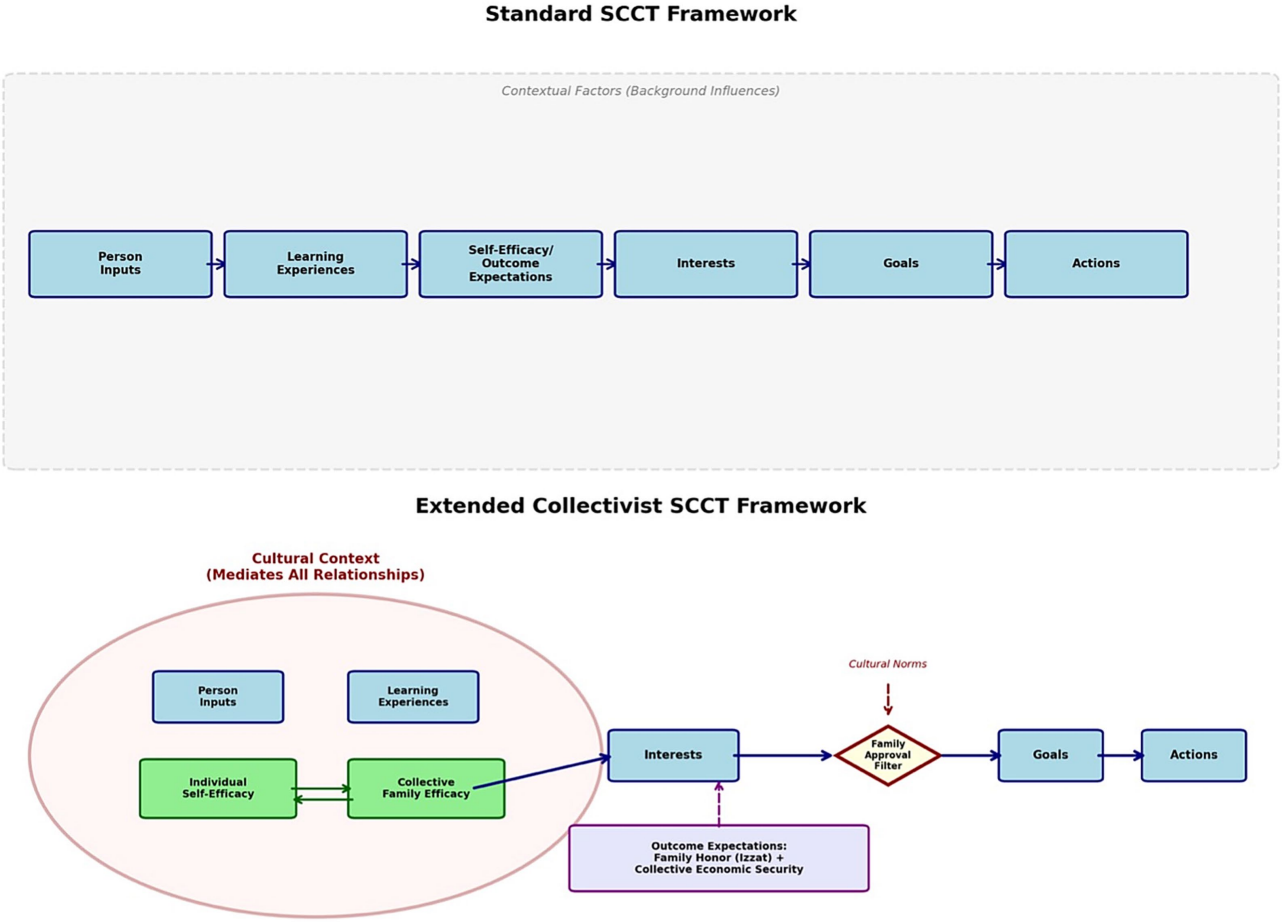
Comparative framework: standard SCCT versus extended collectivist SCCT. Solid arrows (→) = direct causal pathways; dashed arrows (− −>) = cultural/normative influences; bidirectional arrows (⟷) = reciprocal relationships. Gray dashed box = peripheral contextual factors in the Standard SCCT—background influences that moderate but do not fundamentally alter the individual-centric pathway. Rounded rectangles = core constructs; circles = cultural context (mediating environment); diamond = family approval filter (gatekeeping mechanism). Color Coding: Navy/light blue = core SCCT constructs; green = efficacy constructs; purple = outcome expectations; red/pink = cultural context and elements; yellow = family approval filter; gray = peripheral contextual influences (Standard SCCT only). The repositioning of cultural context from peripheral (gray dashed box, top) to central (red circle, bottom) visualizes the theoretical argument that collectivist contexts require a substantive reconceptualization of SCCT rather than mere acknowledgment of cultural moderators. This framework suggests applying SCCT in collectivist contexts requires substantive reconceptualization, not merely acknowledgment of “cultural influences”.

Figure 2 illustrates these extensions, contrasting standard linear SCCT pathways with a collectivist model incorporating collective efficacy, honor-based outcomes, approval-filtered goals, and central cultural embedding.

#### 4.6.2 Communities of practice: theorizing permanent peripherality

CoP theory conceptualizes learning as movement from peripheral participation toward full membership (17, 18). Our findings raise questions about this assumption by demonstrating culturally legitimate exclusion. We identify developmental, structural, and contested peripherality as distinct participation regimes. In Peshawar’s clinical environment, cultural norms render female students legitimately peripheral in male patient care and male students’ peripheral in female patient care. This reframes CoP learning trajectories as socially regulated rather than competence-driven processes, extending identity-based participation critiques (60, 61). Addressing structural peripherality therefore may require institutional redesign rather than learner remediation.

#### 4.6.3 Intersectionality: culturally constructed gender

Intersectionality theory (22, 23, 68, 69) enabled analysis of how gender operated differently across sociocultural contexts. Female identity in Peshawar intersected with honor systems, marital authority structures, and institutional gender segregation policies (12, 14). Many participants explicitly articulated these intersections, demonstrating reflexive awareness of culturally situated constraints (23). Barriers in Peshawar differ qualitatively from Western gender barriers, operating through distinct moral, familial, and institutional logics that require context-specific interventions.

### 4.7 Practical implications

#### 4.7.1 Family centered career counseling

Standard career counseling often targets individual students (66). In contexts where families exercise binding decision-making authority, individual-focused counseling may be insufficient. We propose family centered counseling incorporating family workshops, where parents and students learn about specialty options and work-life integration; family counseling sessions facilitating discussions about interests, obligations, and compromises; counselor training in family systems approaches and cultural gender norms; and marriage-career planning for female students addressing post-marriage career continuation. The challenges include counselor scarcity, counseling stigma, and family time constraints. However, recognizing families as legitimate stakeholders may increase their acceptability.

#### 4.7.2 Culturally engaged gender equity

Standard gender equity interventions emphasize individual empowerment and bias training (26, 32). In contexts where explicit cultural norms structure clinical access, different approaches are necessary.

Geographic Context Matters: Night shift exclusion observed in Peshawar does not represent universal Pakistani practice. Female medical students in Karachi, Lahore, and Islamabad participate in overnight duties during their undergraduate training, without purdah-based restrictions, as observed in Peshawar. This demonstrates that institutional policies restricting female students’ learning access are culturally contingent, not nationally mandated, suggesting that Peshawar institutions could adopt policies that have already been successfully implemented in other Pakistani cities.

Additional approaches include gender-concordant clinical placements guaranteeing female students access to female patients and supervisors, simulation-based learning for gender-restricted experiences (male students learning pelvic exams, female students learning male genitourinary exams, female dental students practicing extractions), female faculty recruitment, particularly in surgical specialties, scheduling redesign creating daytime-intensive learning blocks for students whose families prohibit overnight duties, and explicit policy protection of students’ learning rights regardless of patient preferences.

Challenges include the scarcity of female surgical faculty, expensive simulation equipment, and potential conflicts between student learning rights and patient autonomy principles.

#### 4.7.3 Structured mentorship programs

Given the missing models’ deterrent effects and anti-models’ negative influences, structured mentorship could address social learning gaps (33, 70): specialty-specific longitudinal mentorship (6–12 months); work-life integration discussions where mentors address marriage-career negotiation; culturally coded specialty mentorship pairing students interested in counter-stereotypical specialties with successful practitioners; virtual mentorship when local representation is absent; anti-model mitigation through mentor screening. Challenges include mentor time commitment, ensuring healthy work-life modeling, geographic dispersion and mentor training. However, given the role models’ documented influence on career decisions (34, 35), such investments may prove cost-effective.

#### 4.7.4 Implementation challenges

Recommendations face substantial barriers: resource constraints (simulation equipment, counselor training, faculty recruitment require significant financial investment); cultural resistance (interventions challenging gender norms may face resistance from faculty, families, and students); systemic interdependencies (recruiting female surgical faculty requires conditions where female students feel supported pursuing surgery, yet support requires female faculty; a circular dependency); measurement difficulties (assessing effectiveness requires longitudinal tracking rarely resourced). Addressing these issues requires positioning gender equity as an institutional priority, securing sustained commitment, engaging cultural and religious leaders as allies, and conducting rigorous implementation research (71).

### 4.8 Limitations

This study should be interpreted considering the researchers’ positionality as medical trainees and educators embedded within similar cultural, institutional, and sociolinguistic contexts. While this insider status facilitated participant trust and nuanced interpretation of sensitive topics, it may also have shaped analytic emphasis toward collective family dynamics and gendered constraints. Reflexive memoing, peer debriefing, and team-based analytic discussion were used to mitigate individual interpretive bias; however, complete neutrality is neither possible nor expected in qualitative inquiry, and the findings represent situated interpretations rather than universal claims.

Geographic and cultural specificity: This study focused on four Peshawar institutions characterized by conservative Pashtun cultural norms. Gender-based learning restrictions observed in this setting may not generalize to more liberal Pakistani cities. For example, while female undergraduate medical students in Peshawar were rarely able to participate in night shifts, female students in Karachi, Lahore, and Islamabad routinely participate during undergraduate training. This suggests that gendered learning barriers may operate along a conservatism continuum rather than as uniform national patterns (12, 14). Transferability is therefore highest to similar conservative collectivist contexts, moderate to urban Pakistani settings, and lowest to cosmopolitan South Asian or Western contexts. Comparative multi-city studies would help differentiate national trends from region-specific dynamics. Future comparative work across Pakistani regions (e.g., Peshawar versus Lahore/Multan/Karachi) could additionally examine how locally salient cultural constructs such as *izzat, mahram*-related norms, and masculinity shape clinical participation and specialty trajectories.

Temporal limitations: Interviews were conducted during training, capturing career decision-making in real time; however, post-graduation trajectories were not assessed. Longitudinal follow-up could clarify how specialty trajectories evolve across early adult transitions and changing life projects (72). It would also help determine whether anticipatory specialty screening predicts longer-term outcomes and whether family authority persists as professional identities develop through residency and early practice (1, 2, 73).

Specialty sampling limitations: The sample included relatively few students interested in smaller or emerging specialties. Experiences may differ across subspecialties with distinct training structures or labor market dynamics. Future studies should purposively sample a wider range of specialty pathways to examine whether family influence and gender norms operate similarly across fields.

Single stakeholder perspective: Only student perspectives were captured. The absence of parental, faculty, and residency director viewpoints limits insight into how other stakeholders conceptualize career negotiation. Dyadic or multi-stakeholder designs could better illuminate intergenerational and institutional dynamics.

Social desirability and interviewer effects: Interviews conducted by local medical student researchers may have introduced social desirability bias. While gender-matched interviewing facilitated rapport (47), it may also have constrained disclosure on highly sensitive topics.

Intersectionality depth: Although gender and Pashtun cultural context were central analytic lenses, other intersecting identities received less attention, including socioeconomic status, urban–rural background, sectarian affiliation, disability, and family medical legacy. Wealth may moderate family control by enabling greater autonomy (74). Broader intersectional sampling would strengthen understanding of layered identity effects on career development.

To reduce both social desirability pressures and under-reporting of sensitive identity dimensions, future work could explore hybrid approaches combining insider cultural familiarity with external interviewers (75).

## 5 Conclusion

Clinical rotations shape career decisions through mechanisms inadequately captured by Western-derived career development theories. In collectivist contexts, family negotiation systems, gendered participation norms, and cultural honor frameworks restructure career development processes at their core.

Our theoretical extensions, including collective self-efficacy, honor-based outcome expectations, family approval filters, and central cultural embedding reconceptualize SCCT for collectivist settings. The identification of developmental, structural, and contested peripherality challenges CoP assumptions that competence guarantees participation progression. The “Doctor Daughter” paradox demonstrates culturally specific gender logics requiring intersectional interpretation.

Medical education practices must therefore operate within collectivist frameworks rather than imposing individualistic assumptions. Family-centered counseling culturally engaged gender equity strategies, and structured mentorship represent contextually grounded interventions.

By documenting how family collective agency, structural peripherality, and culturally constructed gender reshape career development, this study demonstrates that global medical education theory must evolve beyond Western individualism. Given the global diversity of medical training contexts, theoretical models must account for collectivist realities rather than marginalizing them as cultural deviations.

## Data Availability

The original contributions presented in the study are included in the article/Supplementary material, further inquiries can be directed to the corresponding authors.
