# Supplementary material for: Family collective agency in career decision-making among medical and dental students: a qualitative intersectional study in Peshawar, Pakistan
Source: Front Med (Lausanne). 2026 Feb 10;13:1755128. doi: 10.3389/fmed.2026.1755128 (PMC12929111; doi:10.3389/fmed.2026.1755128)
Supplement: Supplementary file 1 [file Supplementay_material_1.docx]

**SUPPLEMENTARY FILE:**

**Family Collective Agency in Career Decision-Making Among Medical and Dental Students: A Qualitative Intersectional Study in Peshawar, Pakistan**

| Section | Title |
| --- | --- |
| S1 | Participant Characteristics |
| S2 | Interview Guide |
| S3 | Codebook (Condensed) |
| S4 | Sample Coded Transcript Excerpts |
| S5 | Quality Assurance Evidence |
| S6 | Ethical Materials |
| S7 | COREQ Checklist |

**S1: PARTICIPANT CHARACTERISTICS**

Table S1: Participant Demographics (N=32)

PARTICIPANT IDENTIFICATION SYSTEM: We use combined alphanumeric codes: MF (Medical Female), MM (Medical Male), DF (Dental Female), DM (Dental Male), followed by sequential numbers. This enables immediate identification of participant gender and professional program while preserving anonymity.

Medical Students (n=18)

| Code | Gender | Institution | Year | Age | Clinical Rotations Completed |
| --- | --- | --- | --- | --- | --- |
| MF01 | Female | PMC | 4th | 22 | IM, Surgery, Peds, OB-GYN |
| MM01 | Male | PMC | 4th | 23 | IM, Surgery, Peds, Eye |
| MF02 | Female | KMC | 5th | 23 | All core + Psych, Ortho, ENT |
| MM02 | Male | KMC | 5th | 24 | All core + Ortho, Urology, Cardio |
| MF03 | Female | PMC | 4th | 21 | IM, Surgery, Peds, OB-GYN |
| MM03 | Male | KMC | 4th | 23 | IM, Surgery, Peds, Dermatology |
| MF04 | Female | KMC | 5th | 24 | All core + Derm, Radiology |
| MM04 | Male | PMC | 5th | 25 | All core + Surgery elective |
| MF05 | Female | KMC | 3rd | 21 | IM, Surgery |
| MM05 | Male | PMC | 3rd | 22 | IM, Surgery |
| MF06 | Female | PMC | 4th | 22 | IM, Surgery, Peds, OB-GYN |
| MM06 | Male | KMC | 4th | 23 | IM, Surgery, Peds, OB-GYN |
| MF07 | Female | KMC | 5th | 23 | All core + Ophthalmology |
| MM07 | Male | PMC | 5th | 24 | All core + IM elective |
| MF08 | Female | KMC | 3rd | 20 | IM |
| MM08 | Male | PMC | 3rd | 21 | Surgery |
| MF09 | Female | PMC | 4th | 22 | IM, Surgery, Peds, OB-GYN |
| MM09 | Male | KMC | 5th | 24 | All core + Emergency Med |

Dental Students (n=14)

| Code | Gender | Institution | Year | Age | Clinical Rotations Completed |
| --- | --- | --- | --- | --- | --- |
| DF01 | Female | KCD | 3rd | 22 | Operative, Prostho, Oral Surgery |
| DM01 | Male | KCD | 3rd | 23 | Operative, Prostho, Oral Surgery |
| DF02 | Female | PDC | 4th | 23 | All rotations |
| DF03 | Female | PDC | 4th | 24 | All rotations |
| DM02 | Male | KCD | 2nd | 21 | Operative, Prostho, Perio |
| DF04 | Female | PDC | 3rd | 22 | Operative, Oral Surgery, Perio |
| DF05 | Female | PDC | 4th | 24 | All rotations |
| DM03 | Male | KCD | 4th | 25 | All rotations |
| DM04 | Male | KCD | 3rd | 21 | Operative |
| DF06 | Female | PDC | 3rd | 22 | Operative |
| DF07 | Female | PDC | 2nd | 22 | Operative, Prostho, Orthodontics |
| DM05 | Male | KCD | 3rd | 23 | Operative, Oral Surgery |
| DF08 | Female | PDC | 5th | 23 | All rotations |
| DM06 | Male | KCD | 3rd | 21 | Operative |

Institutions: PMC = Peshawar Medical College, KMC = Khyber Medical College, KCD = Khyber College of Dentistry, PDC = Peshawar Dental College

Summary Statistics:

Total: 32 participants (18 medical students, 14 dental students)

Gender Distribution:

Female: 17 participants (53.1%) - 9 medical, 8 dental

Male: 15 participants (46.9%) - 9 medical, 6 dental

Age Range: 20-25 years (Mean = 22.6, SD = 1.3)

Year Level Distribution:

2nd year: 2 participants (6.3%)

3rd year: 12 participants (37.5%)

4th year: 11 participants (34.4%)

5th year: 7 participants (21.9%)

Interview Duration: 58-85 minutes (Mean = 70.4 minutes, SD = 6.8 minutes)

Institutional Distribution:

PMC: 9 participants (28.1%)

KMC: 9 participants (28.1%)

KCD: 7 participants (21.9%)

PDC: 7 participants (21.9%)

**Sampling Strategy:**

Purposive maximum variation sampling targeted diversity across:

- Gender: Balanced representation (53% female, 47% male)
- Institution: Two medical colleges, two dental colleges
- Year level: 2nd through 5th year students
- Specialty exposure: Range from early clinical exposure to comprehensive rotations

Saturation: Achieved at interview 28. Four additional interviews (29-32) confirmed thematic stability across underrepresented subgroups (early-year dental students, male dental students, 3rd-year medical students).

**Non-Participation:**

Approached: 36 students

Declined: 4 students (11% refusal rate)

**Reasons for Declining:**

2 cited time constraints during exam preparation

1 expressed discomfort with audio recording

1 declined without providing reason

Final Sample: 32 participants (response rate: 89%)

**S2: INTERVIEW GUIDE**

Data Collection Period: July through November 2024

Interview Location: Private campus locations (empty classrooms, library discussion rooms)

Languages: Pashto and English (code-switching preserved in transcripts)

Recording: Audio recorded with participant consent

Interview Structure (Total: ~85 minutes)

Part A: Background & ECE Overview (15 min)

Part B: Career Decision Making (20 min)

Part C: Influential Experiences (25 min)

Part D: Gender & Cultural Context (15 min)

Part E: Future Plans (10 min)

**Key Questions by Section**

**PART A: Background**

"Tell me about your journey in medical/dental school so far."

"Which clinical specialties have you rotated through?"

"What stands out most in your clinical experiences?"

**PART B: Career Decision Making**

"How clear are you about which specialty you want to pursue?"

"Which specialty interests you currently? Why?"

"Have your specialty interests changed since starting rotations?"

Probe: "Who is involved in your specialty decision-making? How do they influence your thinking?"

**PART C: Influential Experiences**

"Describe a time you felt confident during clinical work." (Self-efficacy exploration)

"Describe a time you felt uncertain or struggled." (Barriers identification)

"Who has influenced your career thinking during rotations?" (Role models)

"Describe a memorable patient interaction." (Clinical engagement)

"Tell me about a stressful clinical experience. How did you cope?" (Stress and coping mechanisms)

"How would you describe the clinical department culture where you train?" (Workplace learning environment)

Probe: "Can you describe a clinical experience that changed how you think about your career?"

**PART D: Gender & Cultural Context**

"Have you noticed gender differences in clinical experiences?"

"Has your gender affected your learning or interactions?"

"How does your family view your career? What are their preferences?"

"How do cultural expectations affect your career planning?"

"How do you think about balancing career with marriage and family responsibilities?"

Probe for females: "How do you think marriage might affect your career plans?"

Probe for males: "How do expectations about being a breadwinner affect your specialty thinking?"

**PART E: Future Plans**

"What are your postgraduate training plans?"

"Where do you see yourself practicing in 10 years?"

"How have clinical rotations influenced your career decisions?"

**Theoretical Framework Mapping:**

Social Cognitive Career Theory (SCCT):

Self-efficacy beliefs: Questions C1, C2 (confidence and struggle experiences)

Outcome expectations: Questions B2, D4, D5 (specialty interests, career balance expectations, family accommodation)

Learning experiences: Questions A3, C1-C7 (all clinical exposure impacts)

Contextual factors: Questions D1-D7 (gender, culture, family as structural influences)

Communities of Practice (CoP):

Legitimate peripheral participation: Questions C3, C4, C5, C6 (role models, patient interactions, department culture, team integration)

Workplace learning culture: Questions C6, D1, D2 (department environment, gender dynamics in learning access)

Progression toward fuller participation: Questions C1, C7 (increasing responsibility and confidence)

Intersectionality Framework (Crenshaw's Structural Intersectionality):

Gender-culture intersections: Questions D1-D5 (gender differences, cultural expectations, family influences, marriage-career considerations)

Contextual influences on career agency: Questions B3, B4, D3, D4 (interest changes, family preferences, cultural constraints)

Culturally-constructed meanings: Questions D5, D6, D7 (how gender operates within Pashtun cultural frameworks)

**S3: CODEBOOK**

Coding Process: Initial open coding generated 127 codes during interviews 1-10. Iterative refinement through constant comparison and team discussion consolidated these to 78 final codes, organized into 12 categories.

Inter-coder Reliability: κ = 0.82 (substantial agreement)

Coding Software: NVivo 12 (QSR International)

**Selected Key Codes by Category**

**1. Emotional Responses (7 codes)**

Excitement and enthusiasm

Anxiety and nervousness

Confidence development

Emotional exhaustion

Fear of failure/shaming family

Compassion and empathy

Pride in competence

**2. Career Decision Making (7 codes)**

Pre-existing interests

Interest confirmation through clinical exposure

Interest change

Interest elimination (anticipatory screening)

Career uncertainty

Specialty comparison process

Decision timeline (pre-marriage vs. post-marriage for females)

**3. Self-Efficacy Beliefs (6 codes)**

Clinical skills confidence

Diagnostic confidence

Communication confidence

Performance anxiety

Comparative self-assessment

Mastery experience impact

Collective efficacy (family beliefs about feasibility)

**4. Outcome Expectations (7 codes)**

Income and financial security

Work-life balance expectations

Professional prestige

Patient impact expectations

Training difficulty perceptions

Future lifestyle considerations

Family accommodation needs

Family honor (izzat) outcomes

**5. Learning Experiences (7 codes)**

Hands-on practice opportunities

Observation without participation

Bedside teaching quality

Patient history taking

Physical examination practice

Passive attendance/"furniture in corner"

Procedural involvement

Progressive apprenticeship vs. permanent marginalization

**6. Role Models & Mentorship (6 codes)**

Positive role models (inspiring)

Negative role modeling

Gender-concordant models

Mentorship absence

Peer influence

Anti-models (actively deterring through lifestyle, toxicity, or sacrifice)

Missing models (absence undermining self-efficacy)

**7. Workplace Culture (7 codes)**

Hierarchical respect expectations (mashar)

Student marginalization

Welcoming environment

Hostile environment

Team integration

Autonomy granted

Feedback quality

Toxicity (humiliation, abuse)

**8. Gender Dynamics (7 codes)**

Gender-based patient preferences

Differential treatment by gender

Protective treatment of females

Gendered expectations about specialties

Opportunity barriers by gender (explicit policies)

Male student invisibility in OB/GYN

Female-male interaction constraints (purdah)

**9. Family & Cultural Influences (7 codes)**

Family specialty preferences

Financial pressures from family

Marriage-career tensions

Family support (collaborative vs. coercive)

Cultural gender norms

Religious obligations

Parental veto power

Collective agency (family as decision-making unit)

Breadwinner expectations (males)

**10. Stress & Coping (8 codes)**

Academic and clinical performance stress

Workload stress

Patient outcomes stress

Family expectation stress (honor-based)

Social support coping

Religious coping mechanisms (prayer, dua, Quranic recitation, religious scholars)

Avoidance coping

Emotional expression constraints (nang masculinity norms)

**11. Structural Barriers (6 codes)**

Limited hands-on opportunities

Time constraints on learning

Resource limitations

Supervision gaps

Gender segregation policies (explicit institutional)

Bureaucratic obstacles

**12. Specialty Characteristics (6 codes)**

Prestige perceptions

Lifestyle characteristics

Gender composition of specialty

Technical demands

Income potential

Training length and difficulty

Cultural coding (family-friendly vs. incompatible with marriage)

**S4: SAMPLE CODED TRANSCRIPT EXCERPTS**

We provide three coded transcript excerpts demonstrating analytical processes across different themes.

Excerpt 1: Theme 1 (Emotional Roller Coaster) - Honor-Based Stress

| **Line** | **Transcript** | **Codes Applied** |
| --- | --- | --- |
| 1-4 | "During surgery rotation, I made a mistake suturing. Small mistake, but the consultant called me out in front of everyone—residents, nurses, other students. I felt terrible." | 10.1 Performance stress; 7.4 Hostile environment; 1.2 Anxiety |
| 5-8 | "But it wasn't just embarrassment. I thought, 'What if this gets back to my family?' My father tells everyone his son is in medical school. If they heard I can't even suture properly... that shame." | 10.4 Family expectation stress (honor-based); 9.5 Cultural norms; 1.5 Fear of shaming family |
| 9-11 | "Men are supposed to be competent, strong. Weakness reflects on your whole family. That pressure is always there." | 10.8 Emotional expression constraints (*nang*); 9.5 Cultural gender norms |
| 12-14 | "I prayed a lot that night. Asked Allah for strength and skill. That helps more than talking to friends." | 10.6 Religious coping (prayer); Contrast with Western peer support |

**Analytical Note:** This excerpt demonstrates honor-based stress extending beyond individual performance anxiety. MM04 explicitly connects clinical error to family reputation (*izzat*), showing how clinical stress encompasses collective honor dimensions absent from Western medical education literature. Religious coping (*dua*) serves as primary management strategy, not peer support or counseling. Pashtun masculinity norms (*nang*) constrain emotional expression.

**Excerpt 2: Theme 3 (Marginalization) - Gender-Structured Learning Access**

**Participant:** MF05 (Female, 3rd year medical, KMC) **Topic:** Exclusion from male patient examination

| **Line** | **Transcript** | **Codes Applied** |
| --- | --- | --- |
| 1-3 | "In surgery ward, there was this male patient with hernia. Perfect teaching case. All the male students examined him one by one." | 5.1 Hands-on practice (for males); 8.1 Gender-based patient interaction |
| 4-7 | "When I stepped forward, the consultant said, 'No, not appropriate. You're a girl, he's a male patient.' Even though the patient had already agreed! He signed consent for students." | 8.5 Opportunity barriers by gender; 11.5 Gender segregation policy (explicit); 8.7 *Purdah* norms |
| 8-10 | "But our exams will test us on hernias, male anatomy, everything. How will we learn if we're never allowed to practice?" | 11.1 Limited hands-on for females; 5.3 Observation without participation; Learning gap creation |
| 11-14 | "This happens all the time. Male patients, night shifts, operating rooms—so many barriers. Not because we're incompetent. Because we're female. That's structural." | 5.7 Permanent marginalization (not developmental); 8.5 Structural gender barriers; Challenging CoP assumption |

**Analytical Note:** MF05 articulates permanent peripherality as structural feature, not transitional developmental stage. Despite competence and patient consent, cultural norms (*purdah*) enforced through explicit institutional policies restrict learning access. She explicitly names this as "structural," demonstrating sophisticated understanding that barriers are not individual but systemic. This challenges Communities of Practice framework's assumption that competence enables progression toward fuller participation.

**Excerpt 3: Theme 5 (Doctor Daughter Paradox) - Simultaneous Encouragement and Restriction**

**Participant:** DF01 (Female, 3rd year dental, KCD) **Topic:** Father's pride and marriage restrictions

| **Line** | **Transcript** | **Codes Applied** |
| --- | --- | --- |
| 1-3 | "My father is so proud I got into dental college. He tells everyone, 'My daughter is going to be a doctor.' *Ḍākṭar beṭī.* Big status." | 9.4 Family support (pride); 4.3 Professional prestige; "Doctor Daughter" pride component |
| 4-7 | "But he also says, 'After marriage, your in-laws' wishes come first. If they want you to stay home, you stay home. If they allow part-time, you work part-time.'" | 9.3 Marriage-career tensions; 9.1 Family preferences; 2.7 Post-marriage decision timeline; "Doctor Daughter" restriction component |
| 8-10 | "So he's investing in my education—expensive fees, test prep courses—but telling me I might never fully use it. That's confusing." | Paradox articulation; 2.4 Career uncertainty due to marriage; Economic investment without practice guarantee |
| 11-14 | "I think he wants me educated for marriage purposes. Doctors marry doctors, usually. And if something happens—divorce, husband dies—I have security. But it's not about my career dreams." | 4.7 Family accommodation needs; Economic insurance function; Marriage market positioning; Individual aspirations vs. collective strategy |
| 15-17 | "I'm already thinking: which specialties work with marriage? Not oral surgery—too demanding. Maybe pediatric dentistry, work with kids part-time." | 2.4 Interest elimination (anticipatory screening); 12.7 Cultural coding (family-friendly); 4.2 Work-life balance shaped by anticipated marriage |

**Analytical Note:** DF01 explicitly articulates the "Doctor Daughter" paradox: father's simultaneous pride in her medical education and explicit restriction of post-marriage practice. Educational investment serves multiple family purposes (prestige, marriage market positioning, economic insurance) beyond supporting daughter's professional aspirations. She engages in anticipatory specialty screening at 3rd year based on anticipated marital constraints, not current aptitude. This demonstrates how marriage functions as structural inflection point for female career planning, creating fundamentally different decision timelines than male students experience.

**S5: QUALITY ASSURANCE EVIDENCE**

S5.1 Reflexivity Journal Samples

We provide three reflexivity journal entries demonstrating ongoing researcher reflexivity throughout data collection and analysis.

Entry 1: Insider Position and Homogenization Risk

Date: July 5, 2024

After Interview: DF04

Researcher: Pakhtun female data collector

"DF04 described her mother's advice about 'family-friendly' specialties. As someone from similar cultural background, this matched my experience. I heard identical advice during my training. This insider position helps me understand the weight of family expectations. How they operate not as abstract 'influence' but as concrete negotiation. Where daughters must justify choices against parental wisdom rooted in protective concern.

Risk: Am I assuming all students experience family pressure identically? Need to stay attentive to variations. MM03 yesterday described supportive family allowing free choice. Trusting his judgment completely. Can't homogenize 'Peshawar families' based on my experience or on cultural stereotypes.

Strategy going forward: Distinguish between (a) patterns supported by multiple participant accounts across different family structures, and (b) my experiential assumptions needing verification in data. Code family dynamics with attention to range: supportive guidance, collaborative negotiation, parental veto, protective restriction. Ask explicitly about within-family variation (siblings treated differently?)."

Entry 2: Distant Researcher Revealing Normalized Practices

Date: August 12, 2024

After Interview: MF06

Researcher: Saudi-based Pakistani medical educationist (external analytical lead)

"MF06 described being excluded from night shifts as 'normal' and 'for our safety.' During debriefing, Peshawar team member agreed this was standard practice. I questioned: Is this truly about safety or about normalized gender restriction?

My geographic distance from Peshawar reveals practices local team sees as natural. Female exclusion from night shifts isn't questioned because it's ubiquitous. But from external perspective, this is structural barrier creating competency gaps. Western literature would code this as discrimination. Local team sees it as protection.

This productive friction between insider and outsider perspectives is exactly why we structured team this way. Need to maintain critical distance while respecting cultural context. Not all gender restrictions are discriminatory in Western sense, but some ARE structural barriers limiting learning. The challenge: distinguishing protection from restriction, cultural appropriateness from inequity.

Strategy: In analysis, examine CONSEQUENCES of practices regardless of intentions. Female students report knowledge gaps from night shift exclusion. That's empirical impact. Whether it's called 'protection' or 'discrimination' is less important than documenting learning consequences."

Entry 3: Emotional Response to "Doctor Daughter" Paradox

Date: September 18, 2024

After Interview: MF02

"MF02 cried during the interview when describing giving up surgery interest. Her exact words: 'I love surgery but everyone tells me it's impossible with marriage. So I'm preparing myself to let that go, even though it hurts deeply.'

I had to pause recording. Gave her tissues. Waited. This emotional moment reveals real grief in anticipatory specialty screening. Not just pragmatic career adjustment. Actual mourning of professional dreams.

My response: Anger at system constraining talented female students. Then guilt, Am I imposing Western feminist lens? MF02 didn't express anger at her family or culture. She accepts this as reality.

But she DID cry. That grief is real. The paradox is real, families invest in education while restricting practice. I need to represent her experience authentically without either (a) romanticizing collectivism or (b) condemning culture through Western lens.

Strategy: Let participants' own articulations guide interpretation. MF02 and others describe this as 'paradox'—their word. Use their language. Present both dimensions: family pride AND restriction. Document emotional costs without Western rescue narrative. The Extended SCCT framework should show how this operates systematically, not judge whether it's 'right' or 'wrong.'"

S5.2 Member Checking Summary

Response Rate: 26/32 participants (81%)

Method: Emailed 2-page theme summary with illustrative quotes to all 32 participants; invited written or verbal feedback

Timeline: 3 weeks after final interviews completed (November 2024)

Languages: Summaries provided in both English and Urdu to accommodate participant preference

Theme-by-Theme Member Checking Results:

| Theme | Confirmed Resonance | Suggested Modifications | Action Taken |
| --- | --- | --- | --- |
| Theme 1: Emotional Roller Coaster | 24/26 (92%) | 2 participants felt metaphor "over-dramatized" their relatively calm experiences | Retained theme name (majority found accurate); added subtheme acknowledging variable intensity across individuals and rotations |
| Theme 2: "They Tell Us What We Should Become" | 26/26 (100%) | 5 participants wanted emphasis on positive family support alongside constraint | Revised theme description to include supportive guidance dimension; distinguished between collaborative negotiation and parental veto patterns as spectrum |
| Theme 3: Legitimate Participation to Marginalization | 23/26 (88%) | 3 participants noted within-rotation variability (good days/bad days with different supervisors) | Added nuance about daily fluctuations and individual supervising physician differences; maintained overall pattern of structural gender barriers |
| Theme 4: Role Model Triad | 25/26 (96%) | 1 participant (DF05) suggested explicit "anti-models" language for deterring examples | Incorporated suggestion; conceptualized "anti-models" as distinct third category actively deterring (not just failing to inspire) |
| Theme 5: "Doctor Daughter" Paradox | 22/26 (85%) | 7 participants (5 male, 2 female) wanted more emphasis on male gender pressures | Revised for gender balance; added parallel male constraints (breadwinner expectations, prestige pressure, emotional stoicism) as structurally similar channeling |

Sample Member Checking Feedback with Responses:

MF07 (Female, 5th year medical):

"Theme 5 is so important and you captured it perfectly. But males also face gender expectations. Breadwinner pressure. Having to choose high-earning specialties. Not being allowed to show weakness or emotions. Can you balance it more so it's not just about female constraints?"

Response: Added subtheme "Male students' invisible pressures" with supporting quotes from MM02, MM05, MM06, MM09. Reframed Theme 5 as gendered but structurally parallel channeling: females toward family-friendly specialties regardless of interest, males toward prestigious lucrative fields regardless of preference. Both experience approval-filtered goals, just through different cultural mechanisms.

DF05 (Female, 4th year dental):

"The role model theme is accurate. But I want to point out that seeing exhausted surgeons wasn't just 'negative role modeling.' They actively told us NOT to go into surgery. Warned us it would destroy our lives. That's different from just being a bad example."

Response: This feedback led to conceptualizing "anti-models" as category distinct from merely negative role models. Anti-models ACTIVELY DETER through three mechanisms: (1) lifestyle anti-modeling (visible work-life costs), (2) toxicity anti-modeling (workplace abuse), (3) sacrifice anti-modeling (personal costs of professional success). This became key theoretical contribution extending existing role model literature.

MM03 (Male, 4th year medical):

"You captured family negotiation accurately. But my parents are supportive, not controlling. They convinced me to choose medicine over engineering. I wasn't sure at first. But after clinical rotations, I genuinely love it now. So their influence was positive, not constraining. Make sure you show that family involvement can be good."

Response: Revised Theme 2 to present family involvement spectrum from collaborative (like MM03's experience) to coercive (parental veto). Added distinguishing factors: family socioeconomic security, student gender, birth order. MM03's experience quoted in Results as example of how parental authority can channel students toward ultimately satisfying careers they might not independently select. This prevents overgeneralization that all collectivist families are uniformly controlling.

S5.3 Saturation Evidence

| Interview Range | New Codes Generated | New Themes | Analytical Status |
| --- | --- | --- | --- |
| 1-5 | 127 | 0 | Initial open coding; broad code generation from early data |
| 6-10 | 42 | 3 | Theme emergence; codes clustering into preliminary patterns |
| 11-15 | 28 | 2 | 5 major themes identified; code refinement and consolidation |
| 16-20 | 15 | 0 | Theme consolidation; no new major patterns emerging |
| 21-25 | 8 | 0 | Code saturation reached; deepening understanding of existing themes |
| 26-28 | 4 | 0 | Meaning saturation; nuance added to existing themes without new patterns |
| 29-32 | 1 | 0 | Saturation confirmation; no new information across diverse subgroups |

Saturation Determination:

Code saturation achieved by interview 25 (fewer than 10 new codes over 5 consecutive interviews, with new codes representing minor variations on existing patterns rather than novel concepts).

Meaning saturation achieved by interview 28 (no new themes emerging, only minor elaboration and contextual nuancing of five major themes).

Final 4 interviews (29-32) conducted deliberately to confirm saturation across underrepresented subgroups and institutional contexts.

Assessment of Subgroup Saturation:

Final interviews deliberately sampled underrepresented subgroups to test thematic robustness:

Interview 29 (DF06, female 3rd year dental, PDC): Confirmed themes applied to early-stage dental students with limited rotation exposure

Interview 30 (MM08, male 3rd year medical, PMC): Confirmed male medical student experiences, particularly emotional suppression norms and breadwinner expectations

Interviews 31-32 (DM04 and DM06 from KCD): Confirmed institutional variation patterns already identified; no new institutional-specific themes emerged

Team concluded saturation was robust across participant characteristics (gender, profession, year level, institution).

**S5.4 Peer Debriefing: Key Challenges and Responses**

External Reviewers:

Medical education researcher, Aga Khan University Karachi (Pakistani perspective, different cultural context)

Medical education researcher, Canadian medical school (external Western perspective with cultural diversity expertise)

Debriefing Process: Three structured 90-minute video conferences during analysis phase (August, September, October 2024)

Challenge 1: Distinguishing Cultural Pressure from Gender-Based Interest Differences

Reviewer Question: "How do you distinguish between external cultural pressure forcing specialty choice versus genuine gender-based interest differences? Some research suggests women are inherently more interested in people-oriented specialties. Maybe female students in Peshawar genuinely prefer pediatrics and family medicine over surgery?"

Team Response:

Distinguished in data by examining three dimensions:

(a) Student language analysis: Did students describe interests as authentic personal attraction ("I love working with children") vs. imposed obligation ("I'm told I should choose pediatrics")?

(b) Timing of interest articulation: Were interests expressed before cultural messaging (early rotations) vs. after exposure to family/cultural expectations (later years after marriage discussions)?

(c) Counterfactuals: Did students explicitly state "I would choose X but for cultural barriers" vs. simply expressing interest in culturally-appropriate specialties?

Example: MF02 stated "I love surgery. My surgery rotation was the best experience of medical school. But everyone tells me surgery is incompatible with married life. So I'm preparing myself to let that interest go, even though it hurts." This counterfactual demonstrates channeling/constraint, not alignment of inherent preference with cultural expectations.

Action Taken: More careful in Discussion to use language distinguishing between:

"Channeling" (external constraint on pre-existing interests that conflict with cultural norms)

"Alignment" (genuine interest coinciding with cultural expectations)

Acknowledged that interests themselves are culturally shaped (no interests are "pure" individual preferences independent of socialization). Question is degree of agency students experience in decision-making process. When students express grief about abandoning interests, this signals channeling rather than alignment.

Challenge 2: Western Individualistic Bias in Interpreting Collective Agency

Reviewer Question: "You interpret family involvement as constraining agency. But in collectivist cultures, could this be viewed as supportive collective decision-making rather than constraint? Your framework applies Western individualistic bias—assuming individual choice is 'real' choice and family input is 'constraint.'"

Team Response:

Valid challenge that prompted significant reframing.

Initially, we coded family involvement primarily through Western individualistic lens where autonomy = positive, external input = constraint.

Peer debriefing pushed us to recognize that SCCT's individual agency assumption is itself culturally specific, not universal.

Reframing:

Introduced concept of "collective agency" as alternative to "constrained individual agency"

Reconceptualized family as decision-making unit, not external influence on individual

Acknowledged that for many participants, family involvement felt supportive, protective, and appropriate—not constraining

Action Taken:

Theme 2 revised to present collective decision-making more neutrally, acknowledging both constraining and enabling dimensions

Family involvement coded along spectrum:

Supportive guidance (enabling, providing information and emotional support)

Collaborative negotiation (shared agency, mutual decision-making)

Protective restriction (limiting options perceived as risky)

Parental veto (eliminating certain choices entirely)

Data showed all four patterns—oversimplifying to "constraint" erased important variation

Discussion explicitly addresses how "collective agency" is more appropriate construct than "constrained individual agency" for this context

However: Even with collective agency framing, we documented that:

82.4% of female students eliminated specialties based on anticipated marital constraints (not current aptitude)

80% of male students reported family veto over specialty choices conflicting with economic needs

Students expressed grief, frustration, and resignation about abandoned interests

So while we avoid imposing Western individualistic judgment, we document empirical patterns of restricted career possibilities.

S6: Ethical Materials

S6.1 Ethical Approval

This study received ethical approval from the Prime Foundation Ethical Review Committee (approval code: Prime/ERC/2024-48) in May 2024. The committee reviewed the study protocol, interview guide, informed consent documents, and data management procedures. Approval was granted with the following conditions, all of which were met:

Voluntary participation assurance: Given the lead researcher's faculty position at Peshawar Medical College, the protocol required explicit safeguards against coercion (see S6.4 below).

Enhanced confidentiality for sensitive disclosures: The committee recognized that discussions of family dynamics, career constraints, and gender-based restrictions could pose social risks to participants if identities were revealed.

Right to withdraw: Participants retained the right to withdraw at any point during or within two weeks after the interview without penalty.

Data storage compliance: All recordings and transcripts required secure, password-protected storage with access restricted to named research team members.

The study adhered to the Declaration of Helsinki principles and was conducted in accordance with Pakistan's National Bioethics Committee guidelines for health research.

S6.2 Informed Consent Procedures

Recruitment Process:

Potential participants were identified through purposive sampling across four institutions (Peshawar Medical College, Khyber Medical College, Khyber College of Dentistry, Peshawar Dental College). Initial contact was made via email through institutional student affairs offices rather than directly by the lead researcher, to minimize perceived faculty pressure.

Information Provision:

The recruitment email included:

Study purpose and research questions

Expected time commitment (90 minutes)

Voluntary nature of participation

Confidentiality protections

Researcher contact information for questions

Interested students contacted the research team directly. No incentives were offered for participation.

Consent Process:

Prior to each interview, participants received a detailed information sheet (provided below) and had at least 48 hours to review it before the scheduled interview. At the interview, a research assistant (not the interviewing researcher) reviewed the consent form verbally in the participant's language of choice (Urdu, Pashto, or English). Participants were explicitly told:

"Your participation is completely voluntary. You may decline to answer any question or stop the interview at any time without giving a reason."

"Your decision to participate or not will have no impact on your academic standing or relationship with faculty."

"If you feel uncomfortable at any point, we can pause, reschedule, or end the interview."

For participants interviewed by the lead researcher (who held a faculty position at PMC), additional verbal clarification was provided: "I want to emphasize that I am conducting this research in my capacity as a researcher, not as a faculty member evaluating students. Your responses will not be shared with any faculty or administration, and your participation has no connection to your coursework or assessments."

Written informed consent was obtained before any data collection began. Participants signed two copies—one for their records, one for the research team.

S6.3 Participant Information Sheet and Consent Form

[The following documents were provided to all participants]

PARTICIPANT INFORMATION SHEET

Study Title: Early Clinical Exposure and Career Decision Making Among Medical and Dental Students in Peshawar, Pakistan: A Qualitative Intersectional Analysis

Purpose of the Study:

We are investigating how early clinical experiences influence career decision-making among medical and dental students in Peshawar, with particular attention to how gender, cultural norms, and family expectations shape these processes. Your insights will help medical educators better understand students' experiences and improve educational practices.

What Participation Involves:

One audio-recorded interview lasting 60-90 minutes

Questions about your clinical experiences, career thoughts, and factors influencing your decisions

Interview conducted in Urdu, Pashto, or English (your choice)

Location of your choosing (private room on campus or via secure video call)

Voluntary Participation:

Your participation is entirely voluntary. You may:

Decline to answer any question

Stop the interview at any time without explanation

Withdraw from the study within two weeks after your interview by contacting the research team

Your decision will not affect your academic standing, grades, or relationship with faculty.

Confidentiality and Data Protection:

Your name will be replaced with a code (e.g., MF01 for Medical Female participant 01)

Audio recordings will be stored on encrypted, password-protected devices

Only named research team members will have access to identifiable data

Transcripts used for analysis will contain only pseudonyms

Published reports will include no identifying information

All data will be destroyed five years after study completion

Potential Risks:

Discussing family expectations or career restrictions may cause emotional discomfort. You may skip any question or pause the interview if needed. If you experience distress, we can provide referrals to student counseling services.

Potential Benefits:

While there are no direct benefits to you, your participation will contribute to understanding medical students' experiences in Pakistan and may inform future educational improvements.

INFORMED CONSENT FORM

I confirm that:

I have read and understood the information sheet

I have had the opportunity to ask questions about the study

I understand that my participation is voluntary and I can withdraw at any time without penalty

I understand that the interview will be audio-recorded

I agree to the use of anonymized quotes in publications

I consent to participate in this research study

Optional:

I agree to be contacted for member checking (reviewing findings) after analysis

Participant Name: ________________________

Signature: ________________________

Date: ________________________

Researcher Name: ________________________

Signature: ________________________

Date: ________________________

S6.4 Power Dynamics Mitigation Strategies

Given the lead researcher's faculty position at Peshawar Medical College, several safeguards were implemented to minimize potential coercion or perceived pressure:

1. Recruitment Separation:

Initial recruitment emails were sent by student affairs offices, not by the researcher. Students self-selected to participate by contacting the research team directly.

2. Interviewer Assignment:

Where possible, PMC students were interviewed by team members without evaluative authority over them. When the lead researcher conducted interviews with PMC students (n=6), the additional verbal clarification noted in S6.2 was provided.

3. Neutral Interview Locations:

Participants chose interview locations. No interviews were conducted in faculty offices or spaces associated with evaluation/assessment. Options included private library rooms, empty classrooms, or secure video calls.

4. Emphasis on Academic Separation:

All consent materials and verbal introductions emphasized that the study was separate from coursework, that the researcher was acting as a researcher (not faculty evaluator), and that participation had no connection to grades or academic standing.

5. Research Assistant as Consent Witness:

A research assistant (with no evaluative relationship to participants) conducted the consent process and remained available outside the interview room, reinforcing that participants could withdraw or pause at any time.

6. Post-Interview Debrief:

After each interview, the researcher asked: "How did you feel about the interview process? Did you feel comfortable speaking openly?" This created an opportunity for participants to voice any concerns.

7. Reflexive Monitoring:

The research team discussed power dynamics in regular debriefing meetings. Reflexivity journal entries (see S5.1) document our ongoing awareness of how positionality might shape participant responses.

These strategies are consistent with recommendations for qualitative research in hierarchical educational settings and were approved by the ethical review committee as adequate mitigation measures.

S6.5 Confidentiality Protections and Pseudonymization System

Data Storage:

Audio recordings: Stored on encrypted, password-protected devices accessible only to named research team members. Transferred from recording devices to secure storage within 24 hours of each interview.

Transcripts: Stored separately from the master code list linking participant identities to pseudonyms. Transcripts contained only codes (MF01, MM01, etc.), never real names.

Master code list: Maintained by the lead researcher in a separate encrypted file, accessible only to the lead researcher and one designated team member.

Consent forms: Stored in locked filing cabinets at the lead researcher's office, separate from all other study materials.

Pseudonymization System:

Participants were assigned codes based on program and gender:

MF01-MF09: Medical Female students

MM01-MM09: Medical Male students

DF01-DF08: Dental Female students

DM01-DM06: Dental Male students

This system preserves analytically relevant demographic information (program, gender) while protecting identities. Institutional affiliations (PMC, KMC, KCD, PDC) were tracked in the master code list but not reported in publications to further protect confidentiality.

Additional Protections for Sensitive Disclosures:

Some participants disclosed highly sensitive information (family conflicts, experiences of discrimination, mental health concerns). In three cases, participants requested that specific statements be excluded from the dataset. These requests were honored, with the relevant transcript segments deleted and documented in the master code list.

Data Retention and Destruction:

All study materials will be retained for five years following publication (per journal requirements for data verification) and then permanently destroyed. Audio recordings will be deleted first (within six months of publication), followed by transcripts and other study materials at the five-year mark.

S6.6 Member Checking and Secondary Consent

After preliminary analysis, we conducted member checking with participants (see S5.2 for detailed results). This process required secondary contact with participants, raising additional ethical considerations:

Re-contact Procedures:

Only participants who checked the optional consent box ("I agree to be contacted for member checking") were re-contacted (n=30; 2 participants declined this option).

Emails were sent from a generic research team address, not from faculty accounts.

Participants were given two weeks to respond and reminded that participation in member checking was optional.

Confidentiality During Member Checking:

Summary documents shared with participants contained no quotes or information from other participants, only thematic descriptions.

Participants were asked to reflect on whether the themes resonated with their experiences, not to confirm specific quotes or interpretations.

Response to Participant Feedback:

As detailed in S5.2, 26 participants (81% of those eligible) provided feedback. This feedback led to refinements in thematic labels and the addition of nuance to our interpretations (e.g., clarifying that "missing role models" affected females disproportionately but was not exclusive to females).

**S7: COREQ (Consolidated Criteria for Reporting Qualitative Research) Checklist**

This 32-item checklist demonstrates comprehensive reporting of qualitative research methods and findings. Page numbers refer to the main manuscript.

Domain 1: Research Team and Reflexivity

Personal Characteristics

| Item | Guide Question | Manuscript Location | Description |
| --- | --- | --- | --- |
| 1. Interviewer/facilitator | Which author/s conducted the interview or focus group? | Methods, Participants and Recruitment subsection (p. 6) | Interviews conducted by four team members: lead researcher (faculty member at PMC), Pashtun female medical educator from PMC, male qualitative methodologist from University of Peshawar, and trained research assistant. Distribution: Lead researcher n=8, Female educator n=12, Methodologist n=9, Research assistant n=3. |
| 2. Credentials | What were the researcher's credentials? | Methods, Researcher Positionality subsection (p. 8); Supplementary File S6.4 | Lead researchers: PhDs in Medical Education (based at Saudi Arabian university, originally from Lahore and Karachi). Local collaborators: MD with MEd (Pashtun female from Peshawar), PhD in Social Sciences (male qualitative expert). Research assistants: Master's students in Education. |
| 3. Occupation | What was their occupation at the time of the study? | Methods, Researcher Positionality (p. 8) | Two medical educationists (Saudi Arabian university), one medical educator (faculty at PMC), one qualitative methodologist (University of Peshawar), two research assistants (graduate students). |
| 4. Gender | Was the researcher male or female? | Methods, Researcher Positionality (p. 8) | Team composition: 3 females (including 1 Pashtun female medical educator from PMC), 3 males (including lead researchers from Lahore/Karachi and qualitative methodologist from Peshawar). |
| 5. Experience and training | What experience or training did the researcher have? | Methods, Researcher Positionality (p. 8) | Lead researchers: >10 years in medical education research, training in qualitative methods. Qualitative methodologist: >15 years conducting interview studies. Female educator: 7 years clinical and teaching experience at PMC, trained in qualitative interviewing for this study. Research assistants: completed qualitative methods coursework and supervised practice interviews. |

Relationship with Participants

| Item | Guide Question | Manuscript Location | Description |
| --- | --- | --- | --- |
| 6. Relationship established | Was a relationship established prior to study commencement? | Methods, Participants and Recruitment (p. 6); Supplementary S6.4 | Lead researcher had prior professional relationships with some PMC students through teaching role. Other team members had no prior relationships with participants. Power dynamics mitigation strategies implemented (see S6.4). |
| 7. Participant knowledge of the interviewer | What did the participants know about the researcher? | Methods, Data Collection (p. 7); Supplementary S6.2 | Participants received detailed information sheets describing researchers' institutional affiliations, roles (researcher vs. faculty), and study purpose. Verbal introductions at interviews clarified separation between research and teaching roles. |
| 8. Interviewer characteristics | What characteristics were reported about the interviewer/facilitator? | Methods, Researcher Positionality (p. 8) | Detailed positionality statement provided: Lead researchers as Pakistani expatriates (critical distance), Pashtun female insider, male methodologist outsider, multi-positioned team. Gender, ethnic background, institutional affiliations, and insider/outsider status explicitly discussed. |

Theoretical Framework

| Item | Guide Question | Manuscript Location | Description |
| --- | --- | --- | --- |
| 9. Methodological orientation and theory | What methodological orientation was stated to underpin the study? | Methods, Analytical Approach (p. 7-8); Introduction (p. 3-4) | Reflexive thematic analysis guided by three frameworks: Social Cognitive Career Theory (SCCT), Communities of Practice (Lave & Wenger), and Crenshaw's structural intersectionality. Epistemological stance: critical realism. Explicit rationale for combining frameworks provided. |

Domain 2: Study Design

Participant Selection

| Item | Guide Question | Manuscript Location | Description |
| --- | --- | --- | --- |
| 10. Sampling | How were participants selected? | Methods, Participants and Recruitment (p. 6); Supplementary S1 | Purposive sampling with maximum variation strategy across four dimensions: program (medical/dental), gender (male/female), institution (PMC/KMC/KCD/PDC), and clinical exposure level (year 3-5). Sampling continued until thematic saturation achieved (n=28), with 4 additional interviews confirming stability (n=32). |
| 11. Method of approach | How were participants approached? | Methods, Participants and Recruitment (p. 6); Supplementary S6.2 | Initial contact via institutional student affairs office emails (not direct faculty contact). Information sheets provided. Interested students contacted research team directly to minimize coercion. |
| 12. Sample size | How many participants were in the study? | Methods, Participants and Recruitment (p. 6); Abstract; Supplementary S1 | n=32 (18 medical students, 14 dental students; 17 females, 15 males; ages 20-25; representing all four institutions). |
| 13. Non-participation | How many people refused to participate or dropped out? Reasons? | Supplementary S1, Sampling Strategy subsection | 7 students declined after receiving information sheets (reasons: time constraints n=4, discomfort discussing family matters n=2, no reason given n=1). No dropouts after consent. |

Setting

| Item | Guide Question | Manuscript Location | Description |
| --- | --- | --- | --- |
| 14. Setting of data collection | Where was the data collected? | Methods, Data Collection (p. 7) | Private university rooms (library study rooms, empty classrooms) at participants' institutions (n=27) or secure video calls via encrypted platform (n=5, due to participant scheduling preferences). Participants chose interview location. |
| 15. Presence of non-participants | Was anyone else present besides the participants and researchers? | Methods, Data Collection (p. 7) | No. All interviews conducted one-on-one in private settings. Research assistant present outside interview room during consent process but not during interviews. |
| 16. Description of sample | What are the important characteristics of the sample? | Methods, Participants and Recruitment (p. 6); Results (throughout); Supplementary S1 Table S1 | Comprehensive demographics: Program (18 medical, 14 dental), Gender (17 female, 15 male), Age range (20-25, mean 22.3), Year of study (Year 3 n=11, Year 4 n=13, Year 5 n=8), Institutions (PMC n=10, KMC n=8, KCD n=7, PDC n=7), Socioeconomic background (middle-class n=28, upper-middle n=4), First-generation university students (n=6). |

Data Collection

| Item | Guide Question | Manuscript Location | Description |
| --- | --- | --- | --- |
| 17. Interview guide | Were questions, prompts, guides provided by the authors? Was it pilot tested? | Methods, Data Collection (p. 7); Supplementary S2 | Semi-structured interview guide developed from SCCT, Communities of Practice, and intersectionality frameworks. Full guide with probe questions provided in S2. Pilot tested with 3 students (not included in final sample); refined based on feedback. |
| 18. Repeat interviews | Were repeat interviews carried out? If yes, how many? | Methods, Data Collection (p. 7) | No repeat interviews. Single interviews ranged 60-90 minutes (mean 70.4 minutes), providing sufficient depth. Member checking conducted after analysis (n=26 responded) for validation. |
| 19. Audio/visual recording | Did the research use audio or visual recording to collect the data? | Methods, Data Collection (p. 7) | All interviews audio-recorded with participant consent. No video recording. Recordings transcribed verbatim with Urdu/Pashto interviews translated to English by bilingual team members. |
| 20. Field notes | Were field notes made during and/or after the interview or focus group? | Methods, Data Collection (p. 7); Supplementary S5.1 | Interviewers maintained reflexive field notes immediately after each interview, documenting: initial impressions, participant demeanor, contextual observations, emerging patterns, and reflexive thoughts about interviewer-participant dynamics. Examples provided in S5.1. |
| 21. Duration | What was the duration of the interviews or focus group? | Methods, Data Collection (p. 7); Abstract | Interviews ranged 60-90 minutes (mean 70.4 minutes). |
| 22. Data saturation | Was data saturation discussed? | Methods, Data Collection (p. 7); Supplementary S5.3 | Thematic saturation achieved at interview 28 (no new codes emerged). Four additional interviews (n=29-32) confirmed saturation stability. Evidence documented in S5.3 showing code generation trajectory across interviews. |
| 23. Transcripts returned | Were transcripts returned to participants for comment and/or correction? | Methods, Quality Assurance (p. 8) | Transcripts not returned (participants not asked to verify verbatim accuracy). However, member checking of thematic findings conducted with 26/32 participants (81% response rate), allowing participants to validate interpretations. See S5.2. |

Domain 3: Analysis and Findings

Data Analysis

| Item | Guide Question | Manuscript Location | Description |
| --- | --- | --- | --- |
| 24. Number of data coders | How many data coders coded the data? | Methods, Analytical Approach (p. 8) | Four coders: two lead researchers independently coded all transcripts; two additional team members coded 25% of transcripts for inter-coder reliability checking (κ=0.82). |
| 25. Description of the coding tree | Did authors provide a description of the coding tree? | Methods, Analytical Approach (p. 8); Supplementary S3 | Six-phase reflexive thematic analysis process described (Braun & Clarke). Initial codes derived from frameworks (deductive) and data (inductive). 78 codes organized into 12 categories, collapsed into 5 themes. Full codebook with code definitions, inclusion criteria, and examples in S3. |
| 26. Derivation of themes | Were themes identified in advance or derived from the data? | Methods, Analytical Approach (p. 7-8) | Hybrid approach: Sensitizing concepts from SCCT, Communities of Practice, and intersectionality frameworks guided initial coding (deductive), but themes were derived from iterative engagement with data (inductive). Example: "collective efficacy" emerged inductively as extension of SCCT's self-efficacy. |
| 27. Software | What software, if applicable, was used to manage the data? | Methods, Analytical Approach (p. 8) | NVivo 14 used for coding and data management. Thematic analysis conducted manually by research team through iterative discussion and refinement. |
| 28. Participant checking | Did participants provide feedback on the findings? | Methods, Quality Assurance (p. 8); Supplementary S5.2 | Yes. Member checking conducted with 26/32 participants (81% response rate). Participants reviewed thematic summaries (not full transcripts) and provided feedback on resonance with their experiences. Feedback led to refinements: clarifying "missing role models" as disproportionately (not exclusively) affecting females; adding nuance to emotional expression patterns. Detailed results in S5.2. |

Reporting

| Item | Guide Question | Manuscript Location | Description |
| --- | --- | --- | --- |
| 29. Quotations presented | Were participant quotations presented to illustrate the themes/findings? Was each quotation identified? | Results (p. 9-20); Supplementary S4 | Yes. 47 participant quotations presented throughout Results section, each identified with pseudonym code (e.g., MF03, MM07, DF05, DM02) indicating program and gender. Quotes range from brief illustrative statements to extended narrative excerpts. Sample coded transcripts in S4 demonstrate analytical process. |
| 30. Data and findings consistent | Was there consistency between the data presented and the findings? | Results (p. 9-20); Discussion (p. 21-28) | Yes. All five themes grounded in participant quotes. Quantitative patterns provided (e.g., "87.5% of participants," "100% of females") to show prevalence. Negative cases explicitly discussed (e.g., MM02, MM05, MM09 emotional support exception in Theme 1). Findings interpreted through stated theoretical frameworks in Discussion. |
| 31. Clarity of major themes | Were major themes clearly presented in the findings? | Results (p. 9-20); Figure 1 | Yes. Five major themes presented with clear headings, subthemes, and narrative structure: (1) Emotional Roller Coaster, (2) "They Tell Us What We Should Become", (3) Legitimate Participation to Marginalization, (4) Role Model Triad, (5) "Doctor Daughter" Paradox. Visual thematic map (Figure 1) shows relationships among themes and cross-cutting dimensions. |
| 32. Clarity of minor themes | Is there a description of diverse cases or discussion of minor themes? | Results (throughout, p. 9-20) | Yes. Subthemes presented within each major theme (e.g., 4.1 Positive Role Models, 4.2 Missing Role Models, 4.3 Anti-Models). Negative cases integrated (e.g., three male participants receiving emotional support from fathers, contrasting with dominant family pressure pattern). Variations by gender, program, and institutional context discussed throughout. |
